# Extensive new *Anopheles* cryptic species involved in human malaria transmission in western Kenya

**DOI:** 10.1038/s41598-020-73073-5

**Published:** 2020-09-30

**Authors:** Daibin Zhong, Elizabeth Hemming-Schroeder, Xiaoming Wang, Solomon Kibret, Guofa Zhou, Harrysone Atieli, Ming-Chieh Lee, Yaw A. Afrane, Andrew K. Githeko, Guiyun Yan

**Affiliations:** 1 Program in Public Health, College of Health Sciences, University of California at Irvine, Irvine, CA 92697 USA; 2 Centre for Global Health Research, Kenya Medical Research Institute, Kisumu, Kenya; 3 Department of Medical Microbiology, College of Health Sciences, University of Ghana, Accra, Ghana

**Keywords:** Malaria, Ecological epidemiology, Ecological genetics, Invasive species

## Abstract

A thorough understanding of malaria vector species composition and their bionomic characteristics is crucial to devise effective and efficient vector control interventions to reduce malaria transmission. It has been well documented in Africa that malaria interventions in the past decade have resulted in major changes in species composition from endophilic *Anopheles gambiae* to exophilic *An. arabiensis*. However, the role of cryptic rare mosquito species in malaria transmission is not well known. This study examined the species composition and distribution, with a particular focus on malaria transmission potential of novel, uncharacterized *Anopheles* cryptic species in western Kenya. Phylogenetic analysis based on ITS2 and COX1 genes revealed 21 *Anopheles* mosquito species, including two previously unreported novel species. Unusually high rates of *Plasmodium* sporozoite infections were detected in *An. funestus*, *An. gambiae* and eight cryptic rare species. *Plasmodium falciparum, P. malariae and P. ovale* sporozoite infections were identified with large proportion of mixed species infections in these vectors. This study, for the first time, reports extensive new *Anopheles* cryptic species involved in the malaria transmission in western Kenya. These findings underscore the importance of non-common *Anopheles* species in malaria transmission and the need to target them in routine vector control and surveillance efforts.

## Introduction

Vector control tools, mainly long-lasting insecticide-treated nets (LLIN), indoor residual spraying (IRS), and larval source management (LSM), are key components of malaria control strategies^1^. The massive scale-up of LLINs and IRS in recent years for malaria vector control has led to major changes in biting behaviour, host preference, breeding range, vectorial capacity or vector competence of mosquito vector species in Africa^2^. Vector species-specific differences in ecology and behaviour could substantially affect both malaria transmission and the success of vector control strategies. Accurate identification of malaria vector species and their distribution and bionomics is crucial to devise efficient vector control interventions.

In Africa, most malaria entomological studies focus on the two major vector species complexes *Anopheles gambiae *sensu lato* (s.l.)*, and *An. funestus s.l.*^3–8^. Such studies usually rely on morphological mosquito species identification with keys that could result in morphological misidentifications or discarding unexpected or unknown cryptic sibling species (or rare species)^9^. Cryptic rare species are defined as groups of closely related, but genetically isolated species that are difficult or impossible to distinguish by morphological traits^10^. They are very common in the *Anopheles* genus^9,11–17^. For examples, in southern Africa, Lobo et al. reported 12 of 18 molecularly identified species (including 7 newly identified cryptic species) carrying *Plasmodium* sporozoites using PCR. In West Africa, a cryptic subgroup of *An. gambiae* complex, namely GOUNDRY, which exhibits outdoor resting behaviour, was found to be highly susceptible to *Plasmodium falciparum* infection^18,19^. Additionally, a new species namely *An. fontenillei* in the *An. gambiae* complex was recently discovered in the forested areas of Gabon, Central Africa^20^. In the western Kenya highlands, 17 species of *Anopheles* (including 9 cryptic species) were identified, and nearly half of the cryptic species carried *P. falciparum* parasite DNA^12,14,21^. These studies indicated that many cryptic *Anopheles* species may play an important role in malaria transmission across Africa.

Morphologically indistinguishable cryptic sibling species of mosquitoes are challenges to malaria control programs as a result of incomplete understanding of their biology and their role in malaria transmission. Recent advancements in molecular tools, however, offer unique opportunities for accurate and comprehensive analysis of vector-host-parasite interactions. A number of DNA-based molecular tools have been used to identify cryptic species, including PCR amplification or sequencing of the extrachromosomal mitochondrial DNA (mtDNA) and the ribosomal RNA (rRNA) genes. The rRNA genes have been a preferred target because they are well-studied gene family, and the sequences of certain domains of the genes are very highly conserved among species. Sequencing of rRNA genes has become an important tool for systematic studies of highly diverged taxa^11^. The intergenic spacer (IGS) and internal transcribed spacer 2 (ITS2) region of rRNA gene have been commonly used for development of PCR-based species diagnostic assays and sequence-based cryptic species identifications^9,13,14,16,17,22–25^. The intraspecies ITS2 sequence variation of species in *An. gambiae* complex ranged from 0.07 to 0.43%, whereas interspecies variation ranged between 0.4 and 1.6%^26^. Mitochondrial DNA fragments from the cytochrome oxidase subunit 1 (COX1) gene have been used as DNA barcodes to identify mosquito species^27–29^. However, molecular species identifications based on COX1 alone may have challenges for discriminating between closely related species or species within a complex^30^. Indeed, a recent study indicated that ITS2 provides better resolution than COX1 to differentiate *An. arabiensis* specimens from other *An. gambiae* complex specimens in eastern Ethiopia^31^. Accurate identification of diverse vector species is crucial to devise a tailor-made vector intervention for malaria control based on their specific ecology and behaviour. This is particularly critical as African countries anticipate to achieve malaria elimination from many endemic areas by 2030^32^.

To understand the role of cryptic rare vector species in malaria transmission, the present study examined the composition, distribution, and bionomics of *Anopheles* species in western Kenya. The study also determined *Plasmodium* sporozoite infection status of cryptic rare *Anopheles* species in highland and lowland settings of western Kenya. The multiplex-PCR and species-specific PCR methods were used to identify major vector species and blood meal sources. The ITS2 and the COX1 loci were sequenced and analyzed for cryptic rare *Anopheles* species. Highly sensitive real-time PCR approach was used to detect *Plasmodium* infections in mosquito vectors.

## Results

### Overview of molecular determination of *Anopheles* species

**Figure 1:**
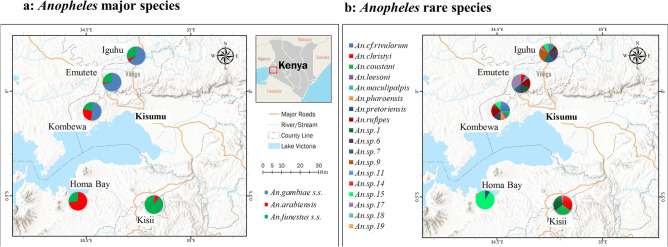
Maps of sampling sites and *Anopheles* species distribution in western Kenya. (**a**) distribution of *Anopheles* major species; (**b**) distribution of *Anopheles* rare species. Pie-chart showed the abundance of *Anopheles* specimens for each site. The maps were generated using ArcGIS Pro 2.6 software. Map source: ESRI, CGIAR, and USGS (available at: www.esri.com).

Out of the 3556 *Anopheles* mosquitoes, 87.1% (3099/3556) were determined by species-specific PCRs or multiplex-PCRs and sequencing as major species *An. gambiae *sensu stricto (hereafter referred to as *An. gambiae*) (1440), *An. arabiensis* (718), and *An. funestus *sensu stricto (hereafter referred to as *An. funestus*) (941) in the five study sites (Fig. 1, Table 1, Supplementary Fig. S1). A subset of 21 randomly selected individuals from each major species identified by PCRs were confirmed by ITS2 sequencing based on similarity (> 98%) to the sequences of anopheline voucher species retrieved from NCBI GenBank database (Supplementary Fig. S2).

**Table 1:** Species composition of *Anopheles* mosquitoes determined by molecular approaches in western Kenya.

Species	ITS2 homology (GenBank no.)	Lowland		Highland			Total
		Homa Bay	Kombewa	Iguhu	Emutete	Kisii	
*An. gambiae*	EU104646	3	295	690	450	2	1440
*An. arabiensis*	KJ522814	480	154	58	14	12	718
*An. funestus*	JN994135	183	116	337	167	138	941
*An. cf.rivulorum*	JN994147		14	3			17
*An. christyi*	GQ870324				14	66	80
*An. coustani*	KJ522815		5	1	1	54	61
*An. leesoni*	KJ522824		3	1		3	7
*An. maculipalpis*	KJ522817				9		9
*An. pharoensis*	KR014827		5				5
*An. pretoriensis*	KJ522820	1	7			1	9
*An. rufipes*	KJ522822		14	2	29	3	48
*An. sp.1*	KJ522813			1	17	52	70
*An. sp.6*	KJ522818			8	17	2	27
*An. sp.7*	KJ522819			8	17	2	27
*An. sp.9*	JN994151			10			10
*An. sp.11*	KJ522823			1		3	4
*An. sp.14*	KJ522826			2			2
*An. sp.15*	KJ522827	11	6	4	1		22
*An. sp.17*	MK043038				56	1	57
*An. sp.18*	Unknown				1		1
*An. sp.19*	Unknown					1	1
Total		678	619	1126	793	340	3556

The remaining 457 collected anophelines (12.9%) were classified into 18 rare species groups based on ITS2 sequence homology. Except for two species groups (*An. sp.18* and *An. sp.19*), the ITS2 sequences of all the species were identified as different species based on their similarity (> 98%) to the sequences of *Anopheles* voucher species retrieved from NCBI GenBank database (Supplementary Fig. S2). The ITS2 sequences of two species could not match with similarity > 98% threshold to reference anopheline sequences or known vector species in GenBank databases, suggesting the existence of novel cryptic species.

**Figure 2:**
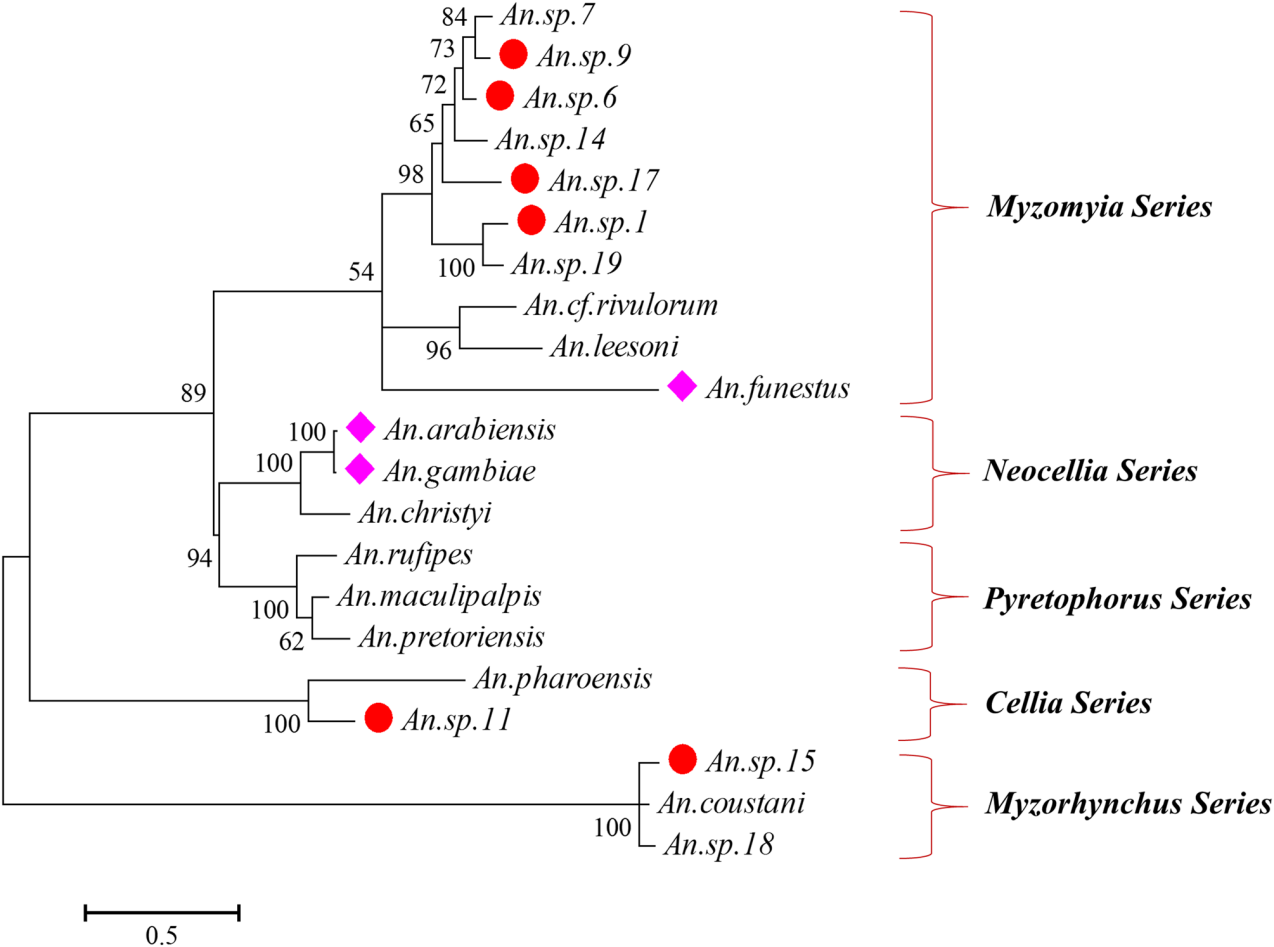
Molecular phylogenetic analysis of ITS2 sequences by Maximum Likelihood method. The phylogenetic tree was constructed using MEGA 7.0 software based on the Kimura 2-parameter model with 1000 bootstrap replicates. Pink filled diamonds showed the major species; red filled circles indicated the novel cryptic species tested positive for *Plasmodium* infections.

Pairwise comparison of ITS2 sequence similarities of the 21 *Anopheles* species indicated that except for one pair with 98.5% identity between *An. gambiae* and *An. arabiensis*, all pairs showed a similarity of 90% or less with confirmed species classifications (Supplementary Table S1). Phylogenetic tree analysis indicated that the 21 species belong to two different Subgenus (Subgenus *Cellia* Theobald and Subgenus *Anopheles* Meigen) in five species series groups, including Myzomyia, Neocellia, Pyretophorus, Cellia, and Myzorhynchus series (Fig. 2, Supplementary Table S2). The two new species *An. sp.18* and *An. sp.19* as well as *An. sp.17* (a recently reported species^13^) belong to two different series groups, and *An. sp.18* belongs to a different Subgenus (Subgenus *Anopheles* Meigen). The ITS2 sequence of *An. sp.9* is homogenous with that of *An. theileri* (GenBank acc. JN994151) and *An. sp. 9 BSL-2014* (GenBank acc. KJ522821)^14^. The ITS2 sequences obtained in the study are available in GenBank with accession numbers: MT408564-MT408584.

### Comparison of morphological and molecular identifications

Of the 3556 mosquitoes tested, 3226 (90.7%) samples with available morphological data were used to evaluate the accuracy of morphological identification as compared to molecular identification. Among the 3226 mosquito samples, 2192 (67.9%) individuals were morphologically identified as *An. gambiae s.l*., 938 (29.1%) as *An. funestus*, 94 (2.9%) as *An. coustani*, and the remaining 2 (0.1%) as *An. pharoensis* (Table 2). The *An. gambiae s.l.* complex and *An. funestus* group (*An. funestus*, *An. cf.rivulorum*, and *An. leesoni*) had a similar percentage of matches (*gambiae* complex: 85.8%, 1881/2192, *funestus* group: 85.2%, 799/938) between molecular assay and morphological identification, while only 53.2% of specimens morphologically identified as *An. coustani* were confirmed by the molecular assay (50/94). Based on molecular assays, *An. gambiae s.l.* complex had the lowest misidentification (4.3%), followed by *An. funestus* group (6.8%), while 18.0% (11/61) *An. coustani* specimens were morphologically misidentified as *An. gambiae* complex (9) or *An. funestus* (2). Based on morphological identification, less than 15% of specimens morphologically assigned to *An. gambiae* complex (14.2%) or *An. funestus* group (14.8%) were identified as other species, while there were 44 specimens morphologically assigned to *An. coustani* (46.8%) that were classified by molecular assay into 9 anopheline species, including *An. rufipes* (17)*, An. funestus* (7)*,* and *An. gambiae* complex (6). Overall, more than 60% (264/427) of the rare species were morphologically misidentified as *An. gambiae s.l.* Specifically, nearly 20% (81/427) and 7.2% (31/427) of rare species were misidentified as *An. funestus* and *An. coustani*, respectively, whereas only 11.7% (50/427) of the rare species were correctly identified as *An. coustani*. Altogether, 84.0% (2710/3226) identification alignment was observed between the morphological and molecular analysis (Table 2).

**Table 2:** Comparison of morphological and molecular identifications in *Anopheles* mosquitoes from western Kenya.

Molecular identification	n	Morphological identification				% of misidentification
		*An. gambiae s.l*	*An. funestus s.l*	*An. coustani*	*An. pharoensis*	
*An. gambiae*	1288	**1216**	66	5	1	4.3^a^
*An. arabiensis*	678	**665**	12	1	0	
*An. funestus*	833	47	**779**	7	0	6.8^b^
*An. cf.rivulorum*	17	1	**14**	2	0	
*An. leesoni*	7	1	**6**	0	0	
*An. coustani*	61	9	2	**50**	0	18.0
*An. christyi*	66	66	0	0	0	100
*An. maculipalpis*	9	6	1	2	0	100
*An. pharoensis*	5	5	0	0	0	100
*An. pretoriensis*	8	7	1	0	0	100
*An. rufipes*	45	27	1	17	0	100
*An. sp.1*	69	62	6	1	0	100
*An. sp.6*	25	9	16	0	0	100
*An. sp.7*	23	6	17	0	0	100
*An. sp.9*	6	4	2	0	0	100
*An. sp.11*	4	3	0	0	1	100
*An. sp.14*	2	0	2	0	0	100
*An. sp.15*	22	8	10	4	0	100
*An. sp.17*	56	48	3	5	0	100
*An. sp.18*	1	1	0	0	0	100
*An. sp.19*	1	1	0	0	0	100
Total	3226	2192	938	94	2	
% of matches		85.8%	85.2%	53.2%	0.0%	
% of misassignment		14.2%	14.8%	46.8%	100%	

Number in bold indicates those individuals identified by both molecular assay and morphological identification. n, total number of individuals identified by molecular assay. ‘misidentification’ means those molecularly determined specimens being morphologically identified as other species; ‘misassignment’ indicates those morphologically assigned specimens being molecularly identified as other species.

^a^*An. arabiensis* and *An. gambiae* were combined as *An. gambiae s.l.* for morphology.

^b^*An. funestus*, *An. cf.rivulorum*, and *An. leesoni* were combined as *funestus* group for morphology.

### Comparison of *Anopheles* species distributions and diversity

Overall, the three major species were found in all five study sites but at varying proportions. *Anopheles funestus* accounted for a large proportion (44.7–98.2%) of species observed throughout the five study sites (Fig. 1A, Table 1). *Anopheles gambiae* was the predominant species in two highland sites (56.7% in Emutete and 61.3% in Iguhu) and one lowland site (47.73% in Kombewa), whereas *An. gambiae* was nearly absent in Homa Bay (0.4%), a lowland site, and Kisii (0.6%), a highland site. *An. arabiensis*, was observed in high proportion in lowland areas (Homa Bay: 70.8% and Kombewa: 24.9%) than in highland areas, which ranged from 1.8% (Emutete) to 5.2% (Iguhu).

Seventeen of 18 rare species were identified in the highland areas, whereas only six rare species were detected in the lowland areas, suggesting that cryptic species might be more related to the sympatric *An. gambiae* than *An. arabiensis*. In lowland sites, the most abundant rare anopheline species was *An. sp.15* (n = 17), followed by *An. rufipes* (n = 14) and *An. cf.rivulorum* (n = 14), whereas multiple rare species (such as *An. christyi, An. sp.1,* and *An. sp.17*) were identified in the highlands (Fig. 1B, Table 1).

A significantly higher species diversity was observed in the highland areas than in the lowland areas (Shannon index *H*, *t*-test, *t* =  − 6.59, *df* = 3419, *p* < 0.001). From 2015 to 2019, the average observed species richness (S) per year was 12.0 ± 0.27 in highland and 6.8 ± 0.25 in lowland (Table 3). In lowland, the highest species abundance (n = 10) was observed in 2018, whereas in highland, the highest species abundance of 15 was detected in 2016 and 2017. A significantly decreased diversity was observed from 2017 to 2018 (Shannon index *H*, *t*-test, *t* = 3.37, *df* = 340, *p* < 0.001) and 2018 to 2019 (Shannon index *H*, *t*-test, *t* = 3.83, *df* = 799, *p* < 0.001) in lowland. However, in highland, a significant increased diversity was observed from 2016 to 2017 (Shannon index *H*, *t*-test, *t* =  − 7.06, *df* = 833, *p* < 0.001), then decreased diversity from 2017 to 2018 (Shannon index *H*, *t*-test, *t* = 1.97, *df* = 787, *p* < 0.05), and remaining high species richness in 2019 (n = 10) (Table 3).

**Table 3:** *Anopheles* species richness and diversity in lowland and highland of western Kenya.

Year	2015	2016	2017	2018	2019
**Highland**					
Individuals	307	819	391	405	337
Observed richness (S)	10	15	15	10	10
Simpson’s dominance (D2)	0.39 (0.36, 0.42)*	0.42 (0.40, 0.44)	0.26 (0.24, 0.29)	0.32 (0.28, 0.36)	0.37 (0.33, 0.42)
Simpson’s diversity (D1)	0.61 (0.58, 0.64)	0.58 (0.56, 0.60)	0.74 (0.71, 0.76)	0.68 (0.64, 0.72)	0.63 (0.58, 0.68)
Shannon’s diversity (H)	1.20 (1.09, 1.31)	1.16 (1.08, 1.24)	1.63 (1.54, 1.73)	1.49 (1.40, 1.58)	1.37 (1.26, 1.48)
Simpson's evenness (€)	0.33 (0.30, 0.37)	0.21 (0.20, 0.23)	0.34 (0.31, 0.38)	0.44 (0.40, 0.49)	0.39 (0.35, 0.44)
Berger–Parker dominance (BP)	0.48 (0.43, 0.54)	0.54 (0.51, 0.58)	0.39 (0.34, 0.44)	0.52 (0.47, 0.56)	0.55 (0.50, 0.61)
**Lowland**					
Individuals	95	180	159	620	243
Observed richness (S)	7	7	7	10	3
Simpson’s dominance (D2)	0.41 (0.33, 0.50)	0.32 (0.30, 0.36)	0.28 (0.26, 0.32)	0.41 (0.38, 0.44)	0.43 (0.39, 0.48)
Simpson’s diversity (D1)	0.59 (0.50, 0.67)	0.68 (0.64, 0.70)	0.72 (0.68, 0.74)	0.59 (0.56, 0.62)	0.57 (0.52, 0.61)
Shannon’s diversity (H)	1.20 (1.01, 1.38)	1.27 (1.15, 1.36)	1.39 (1.31, 1.50)	1.16 (1.09, 1.24)	0.97 (0.89, 1.02)
Simpson's evenness (€)	0.47 (0.40, 0.57)	0.51 (0.48, 0.67)	0.58 (0.53, 0.64)	0.32 (0.30, 0.35)	0.88 (0.81, 0.93)
Berger–Parker dominance (BP)	0.58 (0.47, 0.67)	0.38 (0.36, 0.46)	0.37 (0.31, 0.45)	0.58 (0.54, 0.62)	0.58 (0.52, 0.65)

*The numbers in square brackets represent the 95% confidence interval (CI) for the estimated indices. All these indices were calculated using PAST 4.0 software package.

### Molecular determination of *Anopheles* mosquito host blood meal source

**Figure 3:**
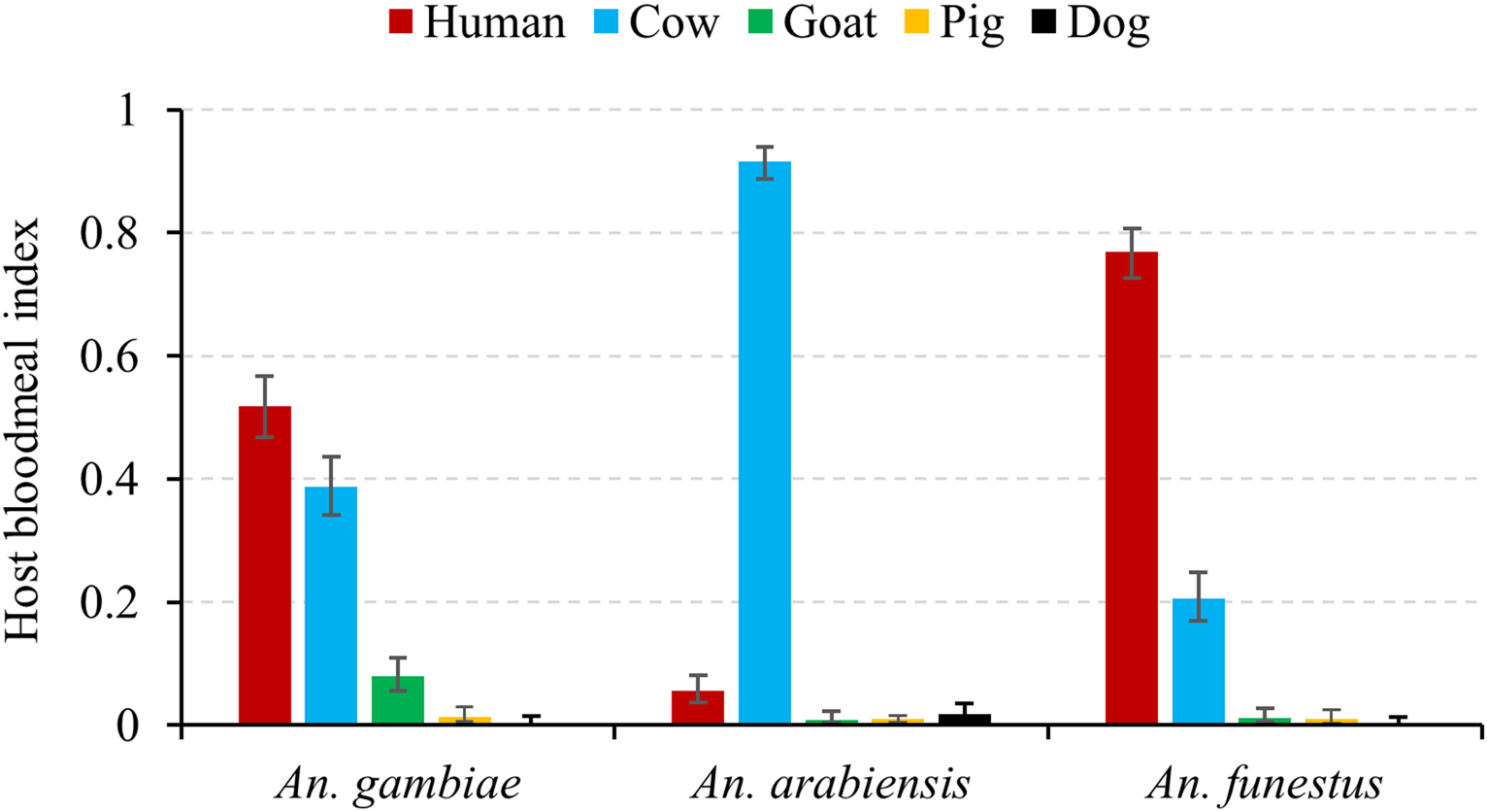
Host blood meal source of Anopheles female mosquitoes in western Kenya. Error bars indicated 95% confidence interval. Bar charts were created in Microsoft Excel 2013 software. Statistical analyses were performed using SAS JMP 14.0 software (SAS Inc., Cary, NC).

A total of 1,372 blood-fed female mosquitoes from 16 *Anopheles* species were successfully genotyped for blood meal sources (Table 4). Of these, 41.6% females were identified to have had human blood meals, 53.6% of mosquito blood meals were identified as bovine, whereas the remained 4.8% individuals had blood meals originating from other animals, e.g., goat, pig, and dog. For the major vector species, the highest human blood index was found in *An. funestus* (0.72), followed by *An. gambiae* (0.51), whereas very few samples of *An. arabiensis* had human blood meals (Pearson's Chi-squared test: χ^2^ = 532.4, *df* = 8, *p* < 0.0001). The majority (> 90%) of *An. arabiensis* blood meals originated from cows (Fig. 3). Similar patterns of blood meal source were observed in the highlands and lowlands. Human blood meal sources were detected in a total of ten rare *Anopheles* species, including eight rare species from highland and two from lowland. The blood meal source of the other three rare species (*An. leesoni, An. maculipalpis,* and *An. pretoriensis*) were identified as bovine (Table 4).

**Table 4:** Number of individuals harbored human bloodmeal source in Anopheles female adults.

Species	Lowland		Highland			Total
	Homa Bay	Kombewa	Iguhu	Emutete	Kisii	
*An. gambiae*		51 (98)*	69 (148)	83 (145)	0 (1)	203 (392)
*An. arabiensis*	19 (372)	2 (39)	1 (10)	1 (6)	1 (5)	24 (432)
*An. funestus*	46 (81)	75 (89)	87 (106)	42 (64)	71 (77)	321 (417)
*An. cf.rivulorum*		1 (11)				1 (11)
*An. christyi*				0 (4)	2 (14)	2 (18)
*An. coustani*					2 (8)	2 (8)
*An. leesoni*		0 (3)	0 (1)		0 (1)	0 (5)
*An. maculipalpis*				0 (3)		0 (3)
*An. pretoriensis*		0 (1)				0 (1)
*An. rufipes*		0 (2)		1 (16)		1 (18)
*An. sp.1*				2 (7)	2 (8)	4 (15)
*An. sp.6*			1 (2)	1 (9)		2 (11)
*An. sp.7*			1 (1)	0 (8)		1 (9)
*An. sp.9*			1 (4)			1 (4)
*An. sp.15*		1 (5)	0 (1)			1 (6)
*An. sp.17*				8 (22)		8 (22)
Total	65 (453)	130 (248)	160 (273)	138 (284)	78 (114)	571 (1372)

*The number in parenthesis represents the total number of blood-fed mosquitoes tested.

### Multiplex-qPCR identification of *Plasmodium* sporozoite infection

**Figure 4:**
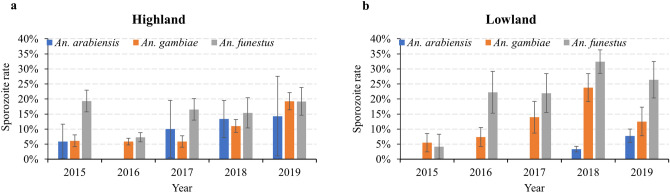
Sporozoite rate of major *Anopheles* mosquito species in western Kenya from 2015 to 2019 (**a**) highland; (**b**) lowland. Error bars represented standard errors of sporozoite rates in the tested samples. Bar charts were created in Microsoft Excel 2013 software. Statistical analyses were performed using SAS JMP 14.0 software (SAS Inc., Cary, NC).

Of the total 3,556 female anopheline mosquitoes tested, 348 (9.8%) were positive for *Plasmodium* sporozoite infections (Table 5). Highest sporozoite rate was observed in *An. funestus* (17.3%; 95% CI 14.9–19.7%), followed by *An. gambiae* (9.9%; 95% CI 8.4–11.4%), and *An. arabiensis* (4.2%; 95% CI 3.6–4.8%). Sporozoite rates were two-fold higher in the lowland than that in highland sites for *An. funestus* (26.4% vs. 13.1%) and 1.5-fold higher in *An. gambiae* (14.3% vs. 7.8%). In contrast, *An. arabiensis* showed a higher sporozoite rate in the highlands (8.3%) compared to the lowlands (3.6%). Surprisingly, *Plasmodium* sporozoites were also detected in eight out of 18 rare anopheline species, confirming the role of rare cryptic species on malaria transmission in western Kenya (Table 5). Between 2015 and 2019, an increasing trend in the annual sporozoite rate was observed in the three major vector species both in the lowland (Chi-squared test: χ^2^ = 9.04, *df* = 1, *p* < 0.01) and highland (Chi-squared test: χ^2^ = 8.08, *df* = 1, *p* < 0.05) study sites (Fig. 4). From the outdoor collection of mosquito samples in 2016 and 2017, four rare species were found positive for *Plasmodium* infection in the two highland sites (Iguhu and Emutete) (Supplementary Table S4). No statistically significant difference in *Plasmodium* infection was detected between indoor and outdoor both in Iguhu (Pearson's Chi-squared test: χ^2^ = 0.78, *df* = 1, *p* = 0.37) and Emutete (Pearson's Chi-squared test: χ^2^ = 0.07, *df* = 1, *p* = 0.78). Three species of *Plasmodium* were detected with the majority being *P. falciparum* (87.6%, 305/348), and the remaining were *P. malarae* and *P. ovale* (Supplementary Table S5). Mixed species infections were common, accounted for 5.7% (20/348) of the total *Plasmodium* sporozoite positive samples. No statistically significant difference between major vector species in the component of parasite species was observed both in lowland (Pearson's Chi-squared test: χ^2^ = 7.35, *df* = 8, *p* = 0.49) and highland (Pearson's Chi-squared test: χ^2^ = 14.84, *df* = 12, *p* = 0.25).

**Table 5:** Number of *Plasmodium* positive mosquitoes detected in *Anopheles* mosquitoes from 2015 to 2019 in western Kenya.

Species	Lowland						Highland					
	2015	2016	2017	2018	2019	Overall	2015	2016	2017	2018	2019	Overall
*An. gambiae*	3 (55)*	5 (68)	6 (43)	20 (84)	6 (48)	40 (298)	9 (148)	26 (445)	9 (153)	23 (209)	36 (187)	103 (1142)
*An. arabiensis*	0 (7)	0 (67)	0 (59)	12 (359)	11 (142)	23 (634)	1 (17)	0 (20)	1 (10)	4 (30)	1 (7)	7 (84)
*An. funestus*	1 (24)	8 (36)	9 (41)	47 (145)	14 (53)	79 (299)	23 (119)	21 (289)	18 (109)	8 (52)	14 (73)	84 (642)
*An. cf.rivulorum*	0 (3)	0 (5)	0 (3)	0 (3)		0 (14)		0 (1)	0 (2)			0 (3)
*An. christyi*									0 (66)		0 (14)	0 (80)
*An. coustani*	0 (1)		0 (1)	0 (3)		0 (5)	0 (1)		0 (9)	1 (45)	0 (1)	1 (56)
*An. leesoni*		0 (2)		0 (1)		0 (3)		0 (2)	0 (1)	0 (1)		0 (4)
*An. maculipalpis*							0 (1)	0 (2)	0 (1)		0 (5)	0 (9)
*An. pharoensis*				0 (5)		0 (5)						
*An. pretoriensis*	0 (1)		0 (1)	0 (6)		0 (8)				0 (1)		0 (1)
*An. rufipes*		0 (1)		0 (13)		0 (14)	0 (2)	1 (17)	0 (2)	0 (6)	0 (7)	1 (34)
*An. sp.1*							0 (3)	2 (6)		0 (53)	0 (8)	2 (70)
*An. sp.6*							0 (8)	1 (11)	1 (2)	0 (6)		2 (27)
*An. sp.7*							0 (8)	0 (8)	0 (9)	0 (2)		0 (27)
*An. sp.9*								1 (10)				1 (10)
*An. sp.11*								1 (1)	0 (3)			1 (4)
*An. sp.14*								0 (2)				0 (2)
*An. sp.15*	0 (4)	0 (1)	0 (11)	0 (1)		0 (17)		1 (4)	0 (1)			1 (5)
*An. sp.17*								0 (1)	3 (22)		0 (34)	3 (57)
*An. sp.18*											0 (1)	0 (1)
*An. sp.19*									0 (1)			0 (1)
Total	4 (95)	13 (180)	15 (159)	79 (620)	31 (243)	142 (1297)	33 (307)	54 (819)	32 (391)	36 (405)	51 (337)	206 (2259)

*The number in parenthesis represents the total number of mosquitoes tested.

### Mitochondrial DNA barcode diversity of cryptic species

**Figure 5:**
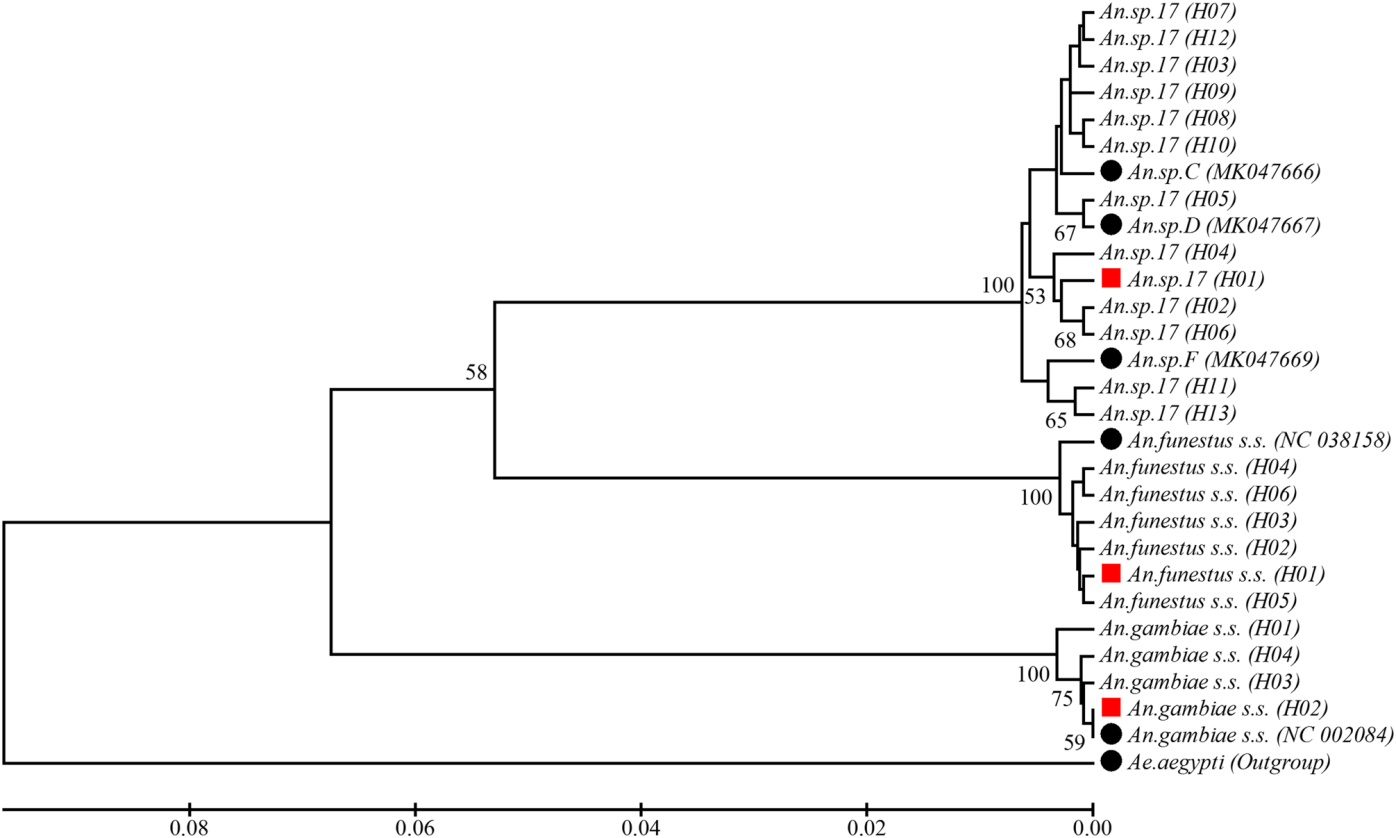
Phylogenetic tree analysis of COX1 haplotypes in the newly discovered *Anopheles* cryptic species in western Kenya. The phylogenetic tree was constructed using MEGA 7.0 software by the bootstrap method with 1000 replications. The red solid squares indicated predominant haplotypes within species, and solid dark circles represented reference sequences retrieved from GenBank at NCBI. The *An. sp.C*, *An. sp.D*, and *An. sp.F* are anopheline cryptic species reported previously in western Kenya (GenBank accession: MK047666, MK047667, and MK047666).

Eighteen of 21 COX1 sequence groups or species had a 1:1 relationship with the ITS2 sequence groups. Two species, *An. sp.18* and *An. sp.19* did not show reliable COX1 sequencing results due to failure of the PCR amplification using the universal COX1 barcoding PCR primers, indicating possible sequence variation in primer regions. However, high COX1 sequence variation was observed in the newly discovered species *An. sp.17* (Supplementary Fig. S2)*.* Among the 21 tested individuals, which showed identical ITS2 gene sequences, 13 haplotypes (*An. sp.17*_H01–*An. sp.17*_H13) of COX1 gene were identified with 17 separating sites. Higher haplotype diversity (*H*d = 0.929, Pi = 0.0081) and nucleotide diversity were found in this cryptic species, as compared to *An. gambiae* (*H*d = 0.700, Pi = 0.0029) and *An. funestus* (*H*d = 0.710, Pi = 0.0015) (n = 21 for each species). Phylogenetic tree analysis indicated that at least four haplotype groups with three of them in different clades matching the reference sequences (*An. sp.C*, *An. sp.D*, and *An. sp.F*, respectively) from NCBI GenBank database (Fig. 5, Supplementary Table S3). No matching references in GenBank was found for the clade with haplotypes *An. sp*.*17*_H01, *An. sp*.*17*_H02, *An. sp.17*_H04, and *An. sp.17*_H06. The sequences obtained in the study were deposited in NCBI GenBank with the accession numbers: MT375202-MT375229.

## Discussion

This study, for the first time, reported eight rare *Anopheles* species with *Plasmodium* sporozoites and human blood meal sources in western Kenya. Although *An. funestus, An. gambiae* and *An. arabiensis* remained as the primary malaria vectors in the region, the presence of 18 cryptic rare *Anopheles* species—half of them being incriminated as malaria vectors—may have serious implications for malaria control program in western Kenya. Mosquito population modification using the innovative vector control techniques, such as gene drives or *Wolbachia* transfection, which rely on mating of mosquitoes to spread, will be unlikely to spread into other reproductively isolated cryptic species except the one (s) in which the tool was introduced. Presence of such diversified vector species coupled with *Plasmodium*-infected outdoor mosquitoes may challenge vector interventions and contribute to malaria transmission stability. Understanding breeding habitats, biting seasonality, resting behaviour and vector competence of these rare vector species is critically required in order to target them in routine malaria control programs.

Phylogenetic tree analysis showed that the seven recently identified and less documented cryptic rare *Anopheles* species along with *An. funestus s.l.* complex belong to *Myzomyia* series group. Among these, four of them (*An. sp.1, An. sp.6, An. sp9,* and *An. sp.17*) were positive for human malaria parasites. Similar findings were reported previously that identified new sibling cryptic *Anopheles* species in western Kenya highlands^13,14^. However, *Plasmodium* infections of the rare species *An. sp.11* and *An. sp.15* are probably the first time being reported in the present study, although these two new species were previously reported without parasite detections^14^. These two rare species were classified into *Cellia* and *Myzorhynchus* series groups, respectively, which encompasses potential vectors, such as *An. pharoensis* and *An. coustani*^9,24^. The present study also for the first time reported two other rare species *An. sp.18* and *An. sp.19* as novel species, since their sequence data were not found in the GenBank database. However, possibly due to a low number of mosquitoes collected, *Plasmodium* infections were not detected in these two species, and no host blood meal sources were found for the samples. An unusually high number of malaria sporozoite rates in rare *Anopheles* species, including *An. sp.1* (2/69) and *An. sp.17* (3/56), suggested their potential role in malaria transmission in western Kenya. Additionally, detecting *Plasmodium* infections from outdoor mosquito collections indicates the presence of outdoor transmission that may not be addressed by indoor-based vector interventions. Further study is however required to characterize breeding habitats, vectorial capacities and biting behaviours of these rare species. Future studies should also investigate environmental factors in the highlands of western Kenya that led to presence of a diverse *Anopheles* species. The potential role of vector competition in the highlands and possible increased availability of breeding sites due to climate change require further study.

A high *Plasmodium* sporozoite rate (nearly 10% or more) for the primary malaria vector species, *An. funestus* and *An. gambiae,* indicated a high level of malaria transmission due to these species in both the highlands and lowlands of western Kenya. Sporozoite rates reported in the present study were over two-fold higher than that of a previous report in western Kenya^33^, probably due to the use of highly sensitive molecular techniques^34^ and multiple parasite species examinations in our study. Similar high sporozoite rates were previously reported for *An. funestus* from elsewhere^9^. These findings generally confirm that *An. funestus* and *An. gambiae,* remain to be the primary malaria vector species in western Kenya. Although these two species are primarily endophagic, the detection of *Plasmodium* infected mosquitoes outdoors might suggest certain behavioural change in their ecology due to continuous use of indoor-based interventions, as reported in neighboring Tanzania^35^. Prolonged and wide-spread use of LLINs could, thereby, favour traits such as biting outdoors or early in the evening. In the southwest Pacific, a study found that high levels of malaria transmission have been maintained by *An. farauti*, a malaria vector that altered its behaviour to blood-feed early in the evening and outdoors and, thereby, avoiding exposure to the insecticides used in IRS^36^. The potential occurrence of such behavioural change in western Kenya requires further study. On the other hand, high sporozoite rate does not necessarily translate to high malaria transmission since disease transmissibility is affected by a number of genetic and environmental factors that determine vector competence^37^. The observed high species diversity in the highland areas may be the result of an increase in new clonal niches that potentially conquered new breeding habitats in the highlands. Unlike *An. funestus* and *An. gambiae*, *An. arabiensis* was a dominant vector species in the lowlands of Homa Bay than highlands of western Kenya. This species is a common vector of malaria in the lowlands of East Africa^38,39^. A low human blood index and low sporozoite rate of *An. arabiensis* in the lowlands indicate that this species primarily tends to feed on non-human blood meal sources, mainly bovine, as reported in Ethiopia^40^. Previous study has suggested that climate change may push *An. arabiensis* to encroach and adapt to the highlands of East Africa^41^.

This study also highlighted the level of misidentifications and misassignments while using morphological keys for species identification. For instance, there were only four species identified using the standard morphological key^42^, while molecular assay revealed the presence of 21 species—suggesting that many *Anopheles* species might be morphologically indistinguishable. The level of misidentification was extremely lower in major species (4–7%) compared to rare species (100%). A number of factors such as level of training and mosquito sample quality determine proper identification of mosquitoes using morphological keys. For instance, morphological identification of *An. coustani* is not complicated due to its unique and conspicuous white patch on its hind legs^42^. Yet, our data indicated that a considerable number of this species was misidentified—suggesting the need for additional training to improve the skill of field entomologists. Overall, species identification using morphological key is affected by a number of factors such as: (1) the quality of the specimen (damaged or intact specimen) when collecting mosquitoes using CDC-LT; (2) morphologically indistinguishable sibling mosquito species; (3) low level of skills on use of morphological keys for species identification by field entomologists. The present study indicated that standard morphological identifications were more reliable for the major species than rare species^21^.

In summary, the present study demonstrated the importance of new *Anopheles* rare species in malaria transmission in western Kenya. Molecular tools help accurately characterize the *Anopheles* vector species in malaria endemic areas. For the first time, eight out of 18 rare *Anopheles* species with *Plasmodium* sporozoites and human blood source were reported in western Kenya, suggesting their secondary role in malaria transmission. Presence of diversified malaria vector species in the highlands might be the consequence of climate change that potentially created new ecological niches for rare vector species, although this requires further investigations. Future studies need to identify and investigate the breeding habitats, and resting and feeding behaviours of these rare vector species in order to devise appropriate vector control strategies that can target all malaria vector species in the region and advance malaria elimination in Africa.

## Methods

### Ethics statement

Ethical approval for the study was obtained from the institutional scientific ethical review board of University of California, Irvine, USA and Maseno University, Kenya. Permission was sought from the chief of each study site. Written informed consent was obtained from heads of the households, and individuals who were willing to participate in the study. All methods used in this study were performed in accordance with the relevant guidelines and regulations.

### Study sites and sample collections

Malaria vector surveillance was conducted in three highland sites (elevation 1500–2300 m) and two lowland sites (1050–1500 m) in western Kenya (Fig. 1). Malaria transmission in highland regions was traditionally regarded as mesoendemic-hyperendemic, unstable, and limited by low temperature, whereas malaria transmission in lowland regions is holoendemic and year around^43,44^. Between 2015 and 2019, adult mosquitoes were collected in five study sites: Iguhu, Emutete, and Kisii in highland regions; and Kombewa and Homa Bay in lowland regions. Indoor-resting mosquitoes were collected by CDC light traps (CDC-LT) and pyrethrum spray catches (PSC) in all study sites. Outdoor-resting mosquitoes were collected by CDC-LT, human landing catches (HLC), clay pot sampling (CP), and pit shelters (PS) sampling methods in Iguhu (2016) and Emutete (2017). These study sites and detailed sampling methods of vector surveillance have been described in previous publications^45–47^. Adult female anopheline mosquitoes were identified to species by morphological keys for the Afrotropical Anophelinae^42,48^. The abdominal status of all individuals was classified into unfed, fed, half gravid and gravid. Mosquito samples were stored individually in 1.5 ml tubes containing desiccated silica gel covered with cotton wool.

### DNA extraction and PCR-based determination of the major species

A large subset of morphologically identified mosquitoes (n = 3556) from the collections above were used for DNA extraction, molecular identification of species, and *Plasmodium* sporozoite infection detections. Female adult mosquitoes were separated individually into head-thoracic portion and abdominal portion for DNA extraction. DNA extraction was performed by using QIAamp 96 DNA QIAcube HT kit on a QIAcube HT 96 automated nucleic acid purification robot (Qiagen, Valencia, CA) according to the manufacturer’s protocol with minor modifications. Specifically, each individual sample was ground in ZR Bashing Bead Lysis tube (2.0 mm beads, Zymo Research Corporation, Irvine, USA) and homogenized in 200 μl lysis solution containing 20 µl of proteinase K (20 mg/ml) using the TissueLyser II system (Qiagen, Hilden, Germany) for 10 min at 30 Hz. The genomic DNA was eluted in a final volume of 100 µl for head-thoracic portion and 200 µl for abdominal portion. For those specimens morphologically identified to *An. gambiae s.l.* complex, multiplex polymerase chain reaction (Multiplex-PCR) was conducted to distinguish *An. gambiae s.s.* and *An. arabiensis* using the species-specific primers (UN/GA/AR) in 28S ribosomal RNA gene according to the method as described by Scott et al.^22^. For those specimens morphologically identified to *An. funestus*, single PCR was conducted to confirm species using the species-specific primers (ITS2A/FUN) in the internal transcribed spacer region (ITS2) on the ribosomal DNA as described by Koekemoer et al.^49^. For the other species and those specimens which failed to amplify by PCR for clear species-specific band or had non-specific or weak amplifications bands^50,51^ using the species-specific primers, additional two PCR amplifications were performed to further identify morphologically misidentified specimens using the primers UN/GA/AR and ITS2A/FUN, respectively.

### DNA sequence-based determination of the rare species

For the specimens which failed to be identified by PCR, and a subset of 21 randomly selected individuals from each major species, additional PCR amplification and DNA sequencing of the ITS2 region of nuclear ribosomal DNA and the COX1 gene were performed using the primer pair ITS2A (TGTGAACTGCAGGACACAT) and ITS2B (TATGCTTAAATTCAGGGGGT) for ITS2^25^, and the primer pair: LCO1490 (GGTCAACAAATCATAAAGATATTGG) and HCO219 (TGATTTTTTGGTCACCCTGAAGTTTA) for COX1^52^. An additional primer pair, 158F (GGTCAACAAATCATAAGGATATTGG) and 890R (GGTCCGAATCCAGGTAAAATTAAA) was designed for COX1 amplification of new species (*An. sp.17*). The PCR was conducted in a total reaction volume of 17 μl, including 1 μl of DNA template (~ 5 ng), 5 pmol of each primer, and 8.5 μl of DreamTaq Green PCR Master Mix (2X) (Thermo Fisher Scientific, Waltham, MA, USA). The thermocycling protocol consisted of an initial activation step of 3 min at 95 °C, followed by 35 amplification cycles of 30 s at 94 °C, 30 s at 55 °C and 45 s at 72 °C, and a final extension step of 6 min at 72 °C. Five microliters of the amplified fragments were examined by electrophoresis on a 1.5% agarose gel. PCR products showing a clear single band on the agarose gel were purified using an enzymatic PCR clean-up technique: 0.3 μl of Exonuclease I (ExoI, 20U/μl) and 1.7 μl of Shrimp Alkaline Phosphtase (SAP, 1U/μl) and 8.0 μl of PCR products. This mixture was incubated at 37 °C for 30 min, followed by 15 min at 80 °C to inactivate the enzymes, then held at 4 °C until sequencing. The premixed samples containing 3 μl of cleaned PCR product, 3 μl of forward primer (10 μM), and 12 μl of ddH2O were sent to Retrogen Inc (San Diego, CA) for sequencing.

### Multiplexed PCR-based blood meal identification

Host blood meal identification of fed mosquitoes was conducted using the multiplexed PCR-based methods as described by Kent et al.^29^, with minor modifications. Specifically, PCR was conducted in a total reaction volume of 13 μl, including 1 μl of DNA template (~ 5 ng), 5 pmol of each primer (Forward primers: Pig573F, Human741F, Goat894F, Dog368F, Cow121F, and Reverse primer UNREV1025), and 6.5 μl of DreamTaq Green PCR Master Mix (2 ×) (Thermo Fisher Scientific, Waltham, MA, USA). The thermocycling protocol consisted of an initial activation step of 3 min at 95 °C, followed by 35 amplification cycles of 30 s at 94 °C, 30 s at 55 °C and 45 s at 72 °C, and a final extension step of 6 min at 72 °C. Five microliters of the amplified fragments were analyzed by 1.5% agarose gel electrophoresis along with 100 bp DNA ladder.

### Multiplexed quantitative PCR (qPCR) assay for *Plasmodium* infections

The DNA extracted from mosquito head-thoracic portion were used for qPCR identification of *Plasmodium* sporozoite infection. An multiplexed real-time qPCR assay was performed by using the published species-specific 18 s ribosomal RNA probes and primers for *Plasmodium falciparum* and *P. malariae*^53^ and *P. ovale*^54^. Multiplexed qPCR was run on QuantStudio 3 Real-Time PCR System (Thermo Fisher Scientific, Carlsbad, CA) in a final volume of 12 µl containing 2 µl of sample DNA, 6 µl of PerfeCTa qPCR ToughMix, Low ROX Master mix (2X) (Quantabio, Beverly, MA), 0.5 µl of each probe (2 µM), 0.4 µl of each forward primers (10 µM), 0.4 µl of each reverse primers (10 µM) and 0.1 µl of double-distilled water. The following temperature profile was applied: hold stage at 50 °C for 2 min and 95 °C for 2 min, followed by 45 cycles of PCR amplification stage at 95 °C for 3 s and 60 °C for 30 s. The standard curve of positive control containing Plasmid DNA (MRA-177 for *P. falciprium*, MRA-179 for *P. malariae*, and MRA-180 for *P. ovale*) from BEI Resources (https://www.beiresources.org) was included in each qPCR plate run with 3 negative controls.

### Statistical analysis

The CodonCode Aligner 9.0.1 (CodonCode Corporation, Centerville, MA) was used to check the sequence quality and trim low-quality bases. BioEdit software^55^ and MView web-based tool^56^ were used to conduct the alignment of the sequences and to calculate pairwise sequence identity and similarity from multiple sequence alignments. A threshold limit of 98% sequence similarity for ITS2 was used to classify sequences into species groups^9^. The consensus sequences within group were compared to the NCBI nr/nt database (https://blast.ncbi.nlm.nih.gov/Blast.cgi). Sequence groups were assigned into known taxa or voucher specimen based on similarity to a voucher specimen sequence at 98% threshold value. The haplotypes of COX1 sequences were compared to both the NCBI nr/nt database and BOLD database (https://www.barcodinglife.org)^29^.

Phylogenetic analyses were performed using Maximum Likelihood (ML) algorithms with the General Time Reversible model for ITS2 consensus sequences of species groups and UPGMA with the Kimura 2-parameter model for COX1 haplotypes in the MEGA version 7.0^57^. The tree nodes were evaluated by bootstrap analysis for 1000 replicates and rooted for COX1 haplotypes using a sequence from *Aedes aegypti* (GenBank: AF390098). The diversity index of Shannon and Simpson, as well as the Simpson’s evenness and dominance index were used to assess *Anopheles* diversity within and between sites. All these indices were calculated using PAST 4.0 software package^58^. To compare diversity between highland and lowland, a *t*-test was used to determine whether they were significantly different^59^.

Human blood index (HBI) is defined as the proportion of freshly fed mosquitoes containing human blood and calculated as described by Garrett-Jones^60^. Mixed blood meals were added to the number of each host blood meals when calculating the HBI separately. Sporozoite rate was estimated by the proportion of the number of positive individuals divided by total number of tested individuals with 95% confidence interval^61^. Statistical analyses were conducted using SAS JMP 14.0 software (SAS Inc., Cary, NC).

## Supplementary information

Supplementary Information.

## Data Availability

The authors declare that the data supporting the findings of this study are available within the paper and its supplementary information file (Supplementary Tables S1–S5; Supplementary Figs. S1, S2).

## References

[1] WHO (2020). Guidelines for Malaria Vector Control.

[2] WHO (2019). World Malaria Report 2019.

[3] Keating J (2005). *Anopheles gambiae s.l.* and *Anopheles funestus* mosquito distributions at 30 villages along the Kenyan coast. J. Med. Entomol..

[4] Noutcha MA, Anumdu CI (2009). Entomological indices of *Anopheles gambiae sensu lato* at a rural community in south-west Nigeria. J. Vector Borne Dis..

[5] Sande S, Zimba M, Chinwada P, Masendu HT, Makuwaza A (2016). Biting behaviour of *Anopheles funestus* populations in Mutare and Mutasa districts, Manicaland province, Zimbabwe: Implications for the malaria control programme. J. Vector Borne Dis..

[6] Ndenga BA (2016). Malaria vectors and their blood-meal sources in an area of high bed net ownership in the western Kenya highlands. Malar. J..

[7] Oyewole IO (2007). Behaviour and population dynamics of the major anopheline vectors in a malaria endemic area in southern Nigeria. J. Vector Borne Dis..

[8] Okara RM (2010). Distribution of the main malaria vectors in Kenya. Malar. J..

[9] Lobo NF (2015). Unexpected diversity of *Anopheles* species in Eastern Zambia: Implications for evaluating vector behavior and interventions using molecular tools. Sci. Rep..

[10] Bickford D (2007). Cryptic species as a window on diversity and conservation. Trends Ecol. Evol..

[11] Collins FH, Paskewitz SM (1996). A review of the use of ribosomal DNA (rDNA) to differentiate among cryptic *Anopheles* species. Insect Mol. Biol..

[12] Conn JE (2016). News from Africa: Novel anopheline species transmit *Plasmodium* in western Kenya. Am. J. Trop. Med. Hyg..

[13] Ogola EO, Chepkorir E, Sang R, Tchouassi DP (2019). A previously unreported potential malaria vector in a dry ecology of Kenya. Parasit. Vectors.

[14] St Laurent B (2016). Molecular characterization reveals diverse and unknown malaria vectors in the western Kenyan highlands. Am. J. Trop. Med. Hyg..

[15] Coetzee M (1994). *Anopheles* crypticus, new species from South Africa is distinguished from *Anopheles coustani* (Diptera: Culicidae). Mosq. Syst..

[16] Hackett BJ (2000). Ribosomal DNA internal transcribed spacer (ITS2) sequences differentiate *Anopheles funestus* and *An. rivulorum*, and uncover a cryptic taxon. Insect Mol. Biol..

[17] Spillings BL (2009). A new species concealed by *Anopheles funestus Giles,* a major malaria vector in Africa. Am. J. Trop. Med. Hyg..

[18] Crawford JE (2016). Evolution of GOUNDRY, a cryptic subgroup of *Anopheles gambiae s.l.*, and its impact on susceptibility to *Plasmodium* infection. Mol. Ecol..

[19] Riehle MM (2011). A cryptic subgroup of *Anopheles gambiae* is highly susceptible to human malaria parasites. Science (New York, N.Y.).

[20] Barrón M (2019). A new species in the major malaria vector complex sheds light on reticulated species evolution. Sci. Rep..

[21] Stevenson J (2012). Novel vectors of malaria parasites in the western highlands of Kenya. Emerg. Infect. Dis..

[22] Scott JA, Brogdon WG, Collins FH (1993). Identification of single specimens of the *Anopheles gambiae* complex by the polymerase chain reaction. Am. J. Trop. Med. Hyg..

[23] Dusfour I (2007). Polymerase chain reaction identification of three members of the *Anopheles sundaicus* (Diptera: Culicidae) complex, malaria vectors in Southeast Asia. J. Med. Entomol..

[24] Norris LC, Norris DE (2015). Phylogeny of anopheline (Diptera: Culicidae) species in southern Africa, based on nuclear and mitochondrial genes. J. Vector Ecol..

[25] Beebe NW, Saul A (1995). Discrimination of all members of the Anopheles punctulatus complex by polymerase chain reaction–restriction fragment length polymorphism analysis. Am. J. Trop. Med. Hyg..

[26] Paskewitz SM, Ng K, Coetzee M, Hunt RH (1993). Evaluation of the polymerase chain reaction method for identifying members of the *Anopheles gambiae* (Diptera: Culicidae) complex in Southern Africa. J. Med. Entomol..

[27] Bourke BP, Oliveira TP, Suesdek L, Bergo ES, Sallum MA (2013). A multi-locus approach to barcoding in the *Anopheles strodei* subgroup (Diptera: Culicidae). Parasit. Vectors.

[28] Kumar NP, Rajavel AR, Natarajan R, Jambulingam P (2007). DNA barcodes can distinguish species of Indian mosquitoes (Diptera: Culicidae). J. Med. Entomol..

[29] Ratnasingham S, Hebert PDN (2007). BOLD: The barcode of life data system. Mol. Ecol. Notes.

[30] Beebe NW (2018). DNA barcoding mosquitoes: Advice for potential prospectors. Parasitology.

[31] Carter TE, Yared S, Hansel S, Lopez K, Janies D (2019). Sequence-based identification of *Anopheles* species in eastern Ethiopia. Malar. J..

[32] WHO (2015). Global Technical Strategy for Malaria 2016–2030.

[33] Githeko AK, Serddvice MW, Mbogo CM, Atieli FK, Juma FO (1993). *Plasmodium falciparum* sporozoite and entomological inoculation rates at the Ahero rice irrigation scheme and the Miwani sugar-belt in western Kenya. Ann. Trop. Med. Parasitol..

[34] Bass C (2008). PCR-based detection of *Plasmodium* in *Anopheles* mosquitoes: A comparison of a new high-throughput assay with existing methods. Malar. J..

[35] Russell TL (2011). Increased proportions of outdoor feeding among residual malaria vector populations following increased use of insecticide-treated nets in rural Tanzania. Malar. J..

[36] Russell TL, Beebe NW, Cooper RD, Lobo NF, Burkot TR (2013). Successful malaria elimination strategies require interventions that target changing vector behaviours. Malar. J..

[37] Pombi M (2018). Unexpectedly high *Plasmodium* sporozoite rate associated with low human blood index in *Anopheles coluzzii* from a LLIN-protected village in Burkina Faso. Sci. Rep..

[38] Kibret S, Wilson GG (2016). Increased outdoor biting tendency of *Anopheles arabiensis* and its challenge for malaria control in Central Ethiopia. Public Health.

[39] Machani MG (2020). Resting behaviour of malaria vectors in highland and lowland sites of western Kenya: Implication on malaria vector control measures. PLoS ONE.

[40] Massebo F, Balkew M, Gebre-Michael T, Lindtjørn B (2015). Zoophagic behaviour of anopheline mosquitoes in southwest Ethiopia: Opportunity for malaria vector control. Parasit. Vectors.

[41] Afrane YA, Githeko AK, Yan G (2012). The ecology of *Anopheles* mosquitoes under climate change: Case studies from the effects of deforestation in East African highlands. Ann. N. Y. Acad. Sci..

[42] Gillies MT, Coetzee M (1987). A supplement to the Anophelinae of Africa south of the Sahara (Afrotropical Region). Publ. S. Afr. Inst. Med. Res..

[43] Shanks GD, Hay SI, Omumbo JA, Snow RW (2005). Malaria in Kenya's western highlands. Emerg. Infect. Dis..

[44] Wanjala CL, Waitumbi J, Zhou G, Githeko AK (2011). Identification of malaria transmission and epidemic hotspots in the western Kenya highlands: Its application to malaria epidemic prediction. Parasit. Vectors.

[45] Ototo EN (2015). Surveillance of malaria vector population density and biting behaviour in western Kenya. Malar. J..

[46] Degefa T (2017). Indoor and outdoor malaria vector surveillance in western Kenya: Implications for better understanding of residual transmission. Malar. J..

[47] Degefa T (2019). Evaluation of the performance of new sticky pots for outdoor resting malaria vector surveillance in western Kenya. Parasit. Vectors.

[48] Gillies MT, Meillon BD (1968). The Anophelinae of Africa south of the Sahara (Ethiopian zoogeographical region). Publ. S. Afr. Inst. Med. Res..

[49] Koekemoer LL, Kamau L, Hunt RH, Coetzee M (2002). A cocktail polymerase chain reaction assay to identify members of the *Anopheles funestus* (Diptera: Culicidae) group. Am. J. Trop. Med. Hyg..

[50] Erlank E, Koekemoer LL, Coetzee M (2018). The importance of morphological identification of African anopheline mosquitoes (Diptera: Culicidae) for malaria control programmes. Malar. J..

[51] Dahan-Moss Y (2020). Member species of the *Anopheles gambiae* complex can be misidentified as *Anopheles leesoni*. Malar. J..

[52] Folmer O, Black M, Hoeh W, Lutz R, Vrijenhoek R (1994). DNA primers for amplification of mitochondrial cytochrome c oxidase subunit I from diverse metazoan invertebrates. Mol. Mar. Biol. Biotechnol..

[53] Veron V, Simon S, Carme B (2009). Multiplex real-time PCR detection of *P. falciparum*, *P. vivax* and *P. malariae* in human blood samples. Exp. Parasitol..

[54] Shokoples SE, Ndao M, Kowalewska-Grochowska K, Yanow SK (2009). Multiplexed real-time PCR assay for discrimination of *Plasmodium* species with improved sensitivity for mixed infections. J. Clin. Microbiol..

[55] Hall TA (1999). BioEdit: A user-friendly biological sequence alignment editor and analysis program for Windows 95/98/NT. Nucl. Acids Symp. Ser..

[56] Brown NP, Leroy C, Sander C (1998). MView: A web-compatible database search or multiple alignment viewer. Bioinformatics.

[57] Kumar S, Stecher G, Tamura K (2016). MEGA7: Molecular evolutionary genetics analysis version 7.0 for bigger datasets. Mol. Biol. Evol..

[58] Hammer Ø, Harper DAT, Ryan PD (2001). PAST: Paleontological statistics software package for education and data analysis. Palaeontol. Electron..

[59] Hutcheson K (1970). A test for comparing diversities based on the Shannon formula. J. Theor. Biol..

[60] Garrett-Jones C (1964). The human blood index of malaria vectors in relation to epidemiological assessment. Bull. World Health Organ..

[61] Newcombe RG (1998). Two-sided confidence intervals for the single proportion: comparison of seven methods. Stat. Med..

