# Supplementary material for: Extensive new Anopheles cryptic species involved in human malaria transmission in western Kenya
Source: Sci Rep. 2020 Sep 30;10:16139. doi: 10.1038/s41598-020-73073-5 (PMC7527330; doi:10.1038/s41598-020-73073-5)
Supplement: Supplementary file 1 — Supplementary Information. [file 41598_2020_73073_MOESM1_ESM.pdf]

Supplementary Data file

Extensive New *Anopheles* Cryptic Species Involved in Human Malaria Transmission in Western Kenya

Daibin Zhong[1*], Elizabeth Hemming-Schroeder[1], Xiaoming Wang[1], Solomon Kibret[1], Guofa Zhou[1], Harrysone Atieli[2], Ming-Chieh Lee[1], Yaw A. Afrane[3], Andrew K. Githeko[2], and Guiyun Yan[1*]

1 Program in Public Health, College of Health Sciences, University of California at Irvine, Irvine, California 92697, USA

2 Centre for Global Health Research, Kenya Medical Research Institute, Kisumu, Kenya

3 Department of Medical Microbiology, College of Health Sciences, University of Ghana, Ghana

*dzhong@uci.edu; guiyuny@uci.edu

Supplementary Table S1 Pairwise comparison of ITS2 sequence similarities of the 21 *Anopheles* species identified in western Kenya

Species	*AR*	*CF*	*CH*	*CO*	*FUN*	*GA*	*LEE*	*MA*	*PH*	*PR*	*RU*	*An.sp.1*	*An.sp.6*	*An.sp.7*	*An.sp.9*	*An.sp.11*	*An.sp.14*	*An.sp.15*	*An.sp.17*	*An.sp.18*	*An.sp.19*
AR	-																				
CF	0.46	-																			
CH	0.74	0.48	-																		
CO	0.34	0.33	0.34																		
FUN	0.31	0.38	0.30	0.32	-																
GA	0.99	0.45	0.75	0.33	0.31	-															
LEE	0.46	0.66	0.47	0.32	0.36	0.46															
MA	0.53	0.45	0.54	0.30	0.29	0.53	0.44	-													
PH	0.23	0.20	0.22	0.22	0.25	0.24	0.20	0.19	-												
PR	0.53	0.46	0.54	0.31	0.28	0.53	0.44	0.84	0.20	-											
RU	0.52	0.47	0.52	0.29	0.30	0.52	0.45	0.77	0.19	0.74	-										
An.sp.1	0.46	0.56	0.46	0.31	0.38	0.45	0.51	0.47	0.20	0.48	0.45	-									
An.sp.6	0.46	0.59	0.48	0.33	0.37	0.46	0.56	0.47	0.20	0.48	0.50	0.72	-								
An.sp.7	0.46	0.57	0.45	0.34	0.37	0.46	0.55	0.47	0.20	0.47	0.49	0.71	0.88	-							
An.sp.9	0.47	0.58	0.46	0.35	0.37	0.47	0.57	0.48	0.21	0.49	0.50	0.71	0.87	0.89	-						
An.sp.11	0.20	0.18	0.19	0.18	0.22	0.20	0.17	0.19	0.49	0.19	0.18	0.17	0.19	0.19	0.18	-					
An.sp.14	0.46	0.58	0.46	0.34	0.38	0.45	0.57	0.48	0.20	0.48	0.49	0.71	0.84	0.81	0.80	0.20					
An.sp.15	0.33	0.32	0.34	0.90	0.33	0.33	0.30	0.29	0.21	0.30	0.29	0.30	0.33	0.33	0.34	0.18	0.33	-			
An.sp.17	0.46	0.59	0.46	0.31	0.39	0.46	0.58	0.48	0.19	0.47	0.49	0.68	0.76	0.75	0.77	0.19	0.72	0.31	-		
An.sp.18	0.29	0.27	0.29	0.85	0.27	0.29	0.28	0.27	0.21	0.27	0.26	0.26	0.29	0.30	0.31	0.17	0.30	0.81	0.27	-	
An.sp.19	0.40	0.48	0.39	0.27	0.32	0.40	0.45	0.40	0.20	0.41	0.39	0.78	0.64	0.63	0.63	0.19	0.64	0.26	0.59	0.29	-

AR, An.arabiensis; CF, An.cf.rivulorum; CH, An.christyi; CO, An.coustani; FUN, An.funestus; GA, An.gambiae; LEE, An.leesoni; MA, An.maculipalpis; PH, An.pharoensis; PR, An.pretoriensis; RU, An.rufipes.

Supplementary Table S2. GenBank accession numbers of ITS2 sequences identified in the 21 *Anopheles* species in western Kenya

Sequence_ID	Species	Isolate	GenBank accession
Seq1	*An. gambiae*	KH01	MT408564
Seq2	*An. arabiensis*	KH02	MT408565
Seq3	*An. funestus*	KH03	MT408566
Seq4	*An. cf.rivulorum*	KH04	MT408567
Seq5	*An. christyi*	KH05	MT408568
Seq6	*An. coustani*	KH06	MT408569
Seq7	*An. leesoni*	KH07	MT408570
Seq8	*An. maculipalpis*	KH08	MT408571
Seq9	*An. pharoensis*	KH09	MT408572
Seq10	*An. pretoriensis*	KH10	MT408573
Seq11	*An. rufipes*	KH11	MT408574
Seq12	*An. sp.1*	KH12	MT408575
Seq13	*An. sp.6*	KH13	MT408576
Seq14	*An. sp.7*	KH14	MT408577
Seq15	*An. sp.9*	KH15	MT408578
Seq16	*An. sp.11*	KH16	MT408579
Seq17	*An. sp.14*	KH17	MT408580
Seq18	*An. sp.15*	KH18	MT408581
Seq19	*An. sp.17*	KH19	MT408582
Seq20	*An. sp.18*	KH20	MT408583
Seq21	*An. sp.19*	KH21	MT408584

Supplementary Table S3. GenBank accession numbers of COX1 haplotype sequences identified in *Anopheles* species of western Kenya

Sequence_ID	Species	Haplotype	GenBank accession
>Seq1	*An. sp.17*	H01	MT375202
>Seq2	*An. sp.17*	H02	MT375203
>Seq3	*An. sp.17*	H03	MT375204
>Seq4	*An. sp.17*	H04	MT375205
>Seq5	*An. sp.17*	H05	MT375206
>Seq6	*An. sp.17*	H06	MT375207
>Seq7	*An. sp.17*	H07	MT375208
>Seq8	*An. sp.17*	H08	MT375209
>Seq9	*An. sp.17*	H09	MT375210
>Seq10	*An. sp.17*	H10	MT375211
>Seq11	*An. sp.17*	H11	MT375212
>Seq12	*An. sp.17*	H12	MT375213
>Seq13	*An. sp.17*	H13	MT375214
>Seq14	*An. funestus*	H01	MT375215
>Seq15	*An. funestus*	H02	MT375216
>Seq16	*An. funestus*	H03	MT375217
>Seq17	*An. funestus*	H04	MT375218
>Seq18	*An. funestus*	H05	MT375219
>Seq19	*An. funestus*	H06	MT375220
>Seq20	*An. gambiae*	H01	MT375221
>Seq21	*An. gambiae*	H02	MT375222
>Seq22	*An. gambiae*	H03	MT375223
>Seq23	*An. gambiae*	H04	MT375224
>Seq24	*An. sp.6*	H01	MT375225
>Seq25	*An. sp.9*	H01	MT375226
>Seq26	*An. cf.rivulorum*	H01	MT375227
>Seq27	*An. maculipalpis*	H01	MT375228
>Seq28	*An. maculipalpis*	H02	MT375229

Supplementary Table S4 Number of *Plasmodium* positive mosquitoes detected indoor and outdoor in western Kenya highlands.

Species	Iguhu (2016)		Emutete (2017)	
	Indoor	Outdoor	Indoor	Outdoor
An. gambiae	10(153)*	7(119)	2(32)	1(14)
An. arabiensis	0(11)	0(2)		0(2)
An. funestus	9(210)	0(14)	5(40)	1(5)
An. cf.rivulorum	0(1)			
An. leesoni	0(1)			
An. maculipalpis				0(1)
An. rufipes	0(2)			0(2)
An. sp.1		0(1)		
An. sp.6	0(3)	0(3)		
An. sp.7	0(6)	0(1)	0(4)	0(4)
An. sp.9	0(5)	1(5)		
An. sp.11		1(1)		
An. sp.14	0(1)	0(1)		
An. sp.15	0(2)	1(2)		0(1)
An. sp.17			2(15)	1(7)
Total	19(395)	10(149)	9(91)	3(36)

* The number in parenthesis represents the total number of mosquitoes tested.

Supplementary Table S5 Species component and proportion of *Plasmodium* infections detected in *Anopheles* mosquitoes in western Kenya.

Species	*pf*	*pm*	*po*	*pf+pm*	*pf+po*	*pm+po*	*pf+pm+po*
An. gambiae	129(90.2)	1(0.7)	8(5.6)	3(2.1)	2(1.4)		
An. arabiensis	26(86.7)		3(10.0)	1(3.3)			
An. funestus	138(84.7)	3(1.8)	8(4.9)	5(3.1)	7(4.3)	1(0.6)	1(0.6)
An. coustani	1(100)						
An. rufipes	1(100)						
An. sp.1	2(100)						
An. sp.6	2(100)						
An. sp.9	1(100)						
An. sp.11	1(100)						
An. sp.15	1(100)						
An. sp.17	3(100)						

* The number in parenthesis represents the percentage of individuals within the mosquito species. *pf, P. falciparum; pm, P. malariae; po, P. ovale.*

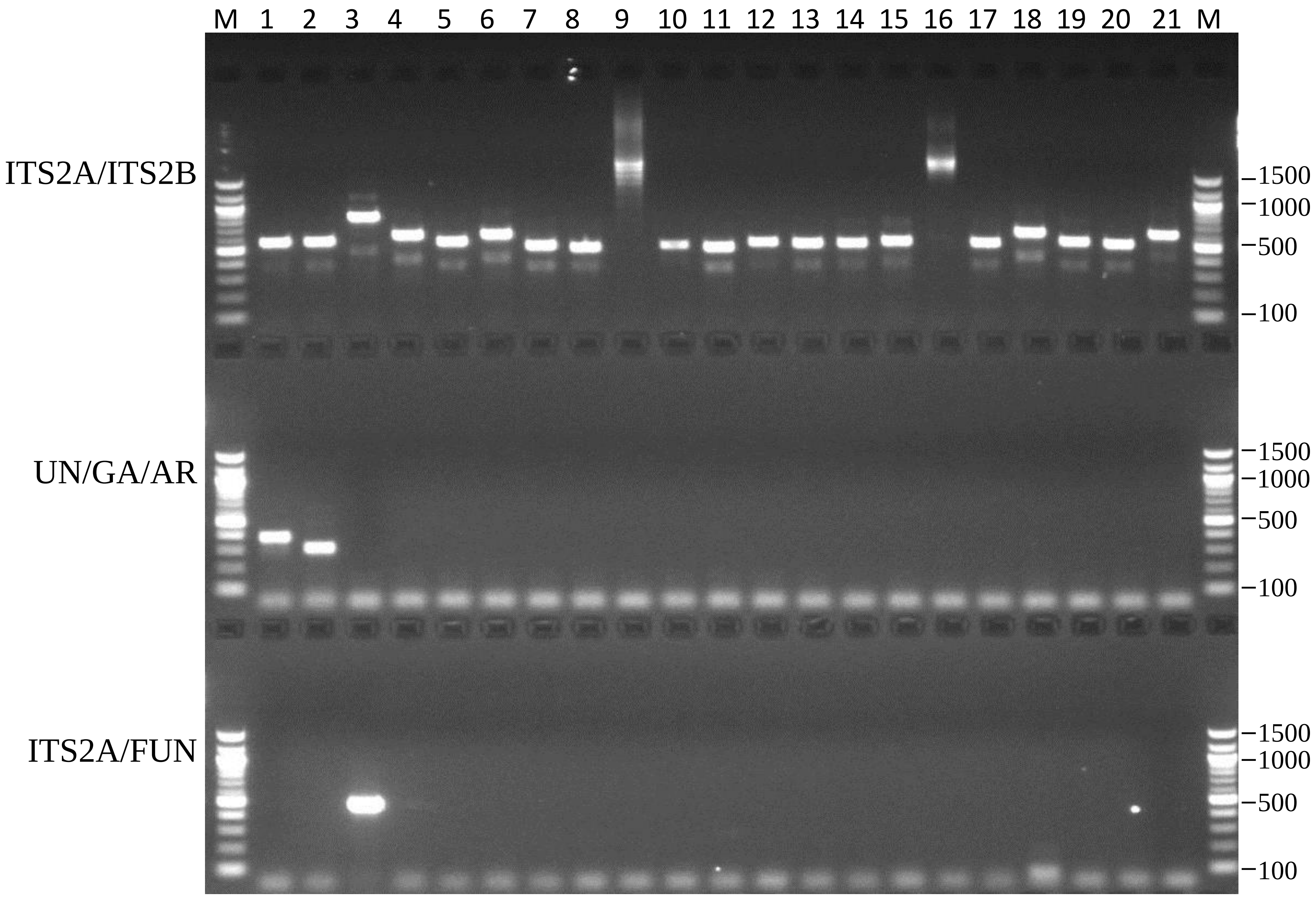

Lane	Species
M	DNA ladder
1	*An. gambiae*
2	*An. arabiensis*
3	*An. funestus*
4	*An. cf.rivulorum*
5	*An. christyi*
6	*An. coustani*
7	*An. leesoni*
8	*An. maculipalpis*
9	*An. pharoensis*
10	*An. pretoriensis*
11	*An. rufipes*
12	*An. sp.1*
13	*An. sp.6*
14	*An. sp.7*
15	*An. sp.9*
16	*An. sp.11*
17	*An. sp.14*
18	*An. sp.15*
19	*An. sp.17*
20	*An. sp.18*
21	*An. sp.19*

Supplementary Fig. S1 Agarose gel electrophoresis of PCR products amplified with the 21 *Anopheles* species in western Kenya. Upper panel, PCR amplification with ITS2A and ITS2B primers for sequencing; Middle panel, PCR amplification with multiplex PCR method with UN/GA/AR primers for identification of *An. gambiae* and *An. arabiensis*; Lower panel, PCR amplification with ITS2A and FUN primer for identification of *An. funestus*. Lane M, 100 bp molecular ladder; Lines 1-21, PCR results of the 21 *Anopheles* species.

Supplementary Fig. S2 shows some examples of multiple sequence alignment comparison for the similarity. (a) ITS2 sequence alignment of *An. funestus*; (b) ITS2 sequence alignment of *An. gambiae*; (c) ITS2 sequence alignment of *An. sp.17*; (d) COX1 sequence alignment of *An. funestus*; (e) COX1 sequence alignment of *An. gambiae*; (f) COX1 sequence alignment of *An. sp.17*. Multiple sequence alignments were performed using BioEdit software (https://bioedit.software.informer.com). The aligned sequences were reformatted by MView web-based tool (http://wavis.img.cas.cz/).

(a)

```
Reference sequence (1): JN994135_An.funestus
Identities normalised by aligned length.
Colored by: identity
                              cov    pid   1 [         .         .         .         .         :         .         .         . 80
1 JN994135_An.funestus 100.0% 100.0%     TGTGAACTGCAGGACACATGAACACCGATAAGTTGAACGCATATTGCGCGTCGGACGATTAAACCCGGCCGATGCACACA
2 MT408566             100.0%  99.6%     TGTGAACTGCAGGACACATGAACACCGATAAGTTGAACGCATATTGCGCGTCGGACGATTAAACCCGGCCGATGCACACA

                              cov    pid  81           .         1         .         .         .         .         :         . 160
1 JN994135_An.funestus 100.0% 100.0%     TTCTTGAGTGCCTATCAATTCCTTGATATACAACAAACCAAACTTCAGGGTGGAGCGTGCCACAATAGAACACTATGGCG
2 MT408566             100.0%  99.6%     TTCTTGAGTGCCTATCAATTCCTTGATATACAACAAACCAAACTTCAGGGTGGAGCGTGCCACAATAGAACACTATGGCG

                              cov    pid 161           .         .         .         2         .         .         .         . 240
1 JN994135_An.funestus 100.0% 100.0%     AGCAGCCCGTCTAGTGTCGTGGGGGAAACACGCTTCCACACTGTGCATAATGGCGTGCTCGGGACCTTTGTTGGGACCGC
2 MT408566             100.0%  99.6%     AGCAGCCCGTCTAGTGTCGTGGGGGAAACACGCTTCCACACTGTGCATAATGGCGTGCTCGGGACCTTTGTTGGGACCGC

                              cov    pid 241           :         .         .         .         .         3         .         . 320
1 JN994135_An.funestus 100.0% 100.0%     AGGGCGCTGAAAGTAAAGGGGTGAACCGCATAAATCGCACGCACGTAAACGCGCACACACACAAATAGAGTGAGACGTAT
2 MT408566             100.0%  99.6%     AGGGCGCTGAAAGTAAAGGGGTGAACCGCATAAATCGCACGCACGTAAACGCGCACACACACAAATAGAGTGAGACGTAT

                              cov    pid 321           .         .         :         .         .         .         .       4 400
1 JN994135_An.funestus 100.0% 100.0%     CGTAGGATACCGCTAAGAGTACGTTGTGAAACATGGGGAAATTCAATCGAAAACCTCTTTGATGTCCAAGATTTCGTTGA
2 MT408566             100.0%  99.6%     CGTAGGATACCGCTAAGAGTACGTTGTGAAACATGGGGAAATTCAATCGAAAACCTCTTTGATGTCCAAGAATTCGTTGA

                              cov    pid 401           .         .         .         .         :         .         .         . 480
1 JN994135_An.funestus 100.0% 100.0%     CCGTATCCGTCGTAATACTGGATCAACGTGCTTGGGGGAAAACGTCAAAGGGTTTTATAATAGTGGTGCATGATTAACCC
2 MT408566             100.0%  99.6%     CCGTATCCGTCGTAATACTGGATCAACGTGCTTGGGGGAAAACGTCAAAGGGTTTTATAATAGTGGTGCATGATTRACCC

                              cov    pid 481           .         5         .         .         .         .         :         . 560
1 JN994135_An.funestus 100.0% 100.0%     ATCGATGCCCGAGGGGAACATGTTGTCCAATACAATAGTGGTGCAGTTGGCTCGACATGCTCGGGGGGAGACATCGTGGG
2 MT408566             100.0%  99.6%     ATCGATGCCCGAGGGGAACATGTTGTCCAATACAATAGTGGTGCAGTTGGCTCGACATGCTCGGGGGGAGACATCGTGGG

                              cov    pid 561         ] 570
1 JN994135_An.funestus 100.0% 100.0%     TCCAAGTCGA
2 MT408566             100.0%  99.6%     TCCAAGTCGA
```

(b)

```
Reference sequence (1): EU104646_An.gambiae
Identities normalised by aligned length.
Colored by: identity
                          cov    pid   1 [         .         .         .         .         :         .         .         . 80
1 EU104646_An.gambiae 100.0% 100.0%     TGTGAACTGCAGGACACATGAACATTGATAAGTTGAACGCATATGGCGCATCGGACGTTTAATCCCGACCGATGCACACA
2 MT408564            100.0%  99.8%     TGTGAACTGCAGGACACATGAACATTGATAAGTTGAACGCATATGGCGCATCGGACGTTTAATCCCGACCGATGCACACA

                          cov    pid  81           .         1         .         .         .         .         :         . 160
1 EU104646_An.gambiae 100.0% 100.0%     TTCTTGAGTGCCTACTAATTACCAAAGTCTCATTTAGTTAACTACAGTGGCCGTCCGCGAAGGTGCCCGGGTCATCCGAC
2 MT408564            100.0%  99.8%     TTCTTGAGTGCCTACTAATTACCAAAGTCTCATTTAGTTAACTACAGTGGCCGTCCGCGAAGGTGCCCGGGTCATCCGAC

                          cov    pid 161           .         .         .         2         .         .         .         . 240
1 EU104646_An.gambiae 100.0% 100.0%     GCACTGGGCGGTCGCTGTGCATAATGACGTGCTTGGTCCCCGTCTGCGGGTCCTCGGGCGTTGAAAGTGGACACTCTCGA
2 MT408564            100.0%  99.8%     GCACTGGGCGGTCGCTGTGCATAATGACGTGCTTGGTCCCCGTCTGCGGGTCCTCGGGCGTTGAAAGTGGACACTCTCGA

                          cov    pid 241           :         .         .         .         .         3         .         . 320
1 EU104646_An.gambiae 100.0% 100.0%     GCGTATGTTGGATGCGTTTCGTGTTGGTGGTGTTTGATGCGTAGGGCTTGTGGTGTGTGTCAAGCCGCATGGTTCGAACT
2 MT408564            100.0%  99.8%     GCGTATGTTGGATGCGTTTCGTGTTGGTGGTGTTTGATGCGTAGGGCTTGTGGTGTGTGTCAAGCCGCATGGTTCGAACT

                          cov    pid 321           .         .         :         .         .         .         .         4 400
1 EU104646_An.gambiae 100.0% 100.0%     AATGCTACGTCGTTCCCGATGGCCACCGGCAGTCTACTCTCCAGGCTAAAGTCGGCTCGTCTAGGGATTCGGAAAGCTAA
2 MT408564            100.0%  99.8%     AATGCTACGTCGTTCCCGATGGCCACCGGCAGTCTACTCTCCAGGCTAAAGTCGGCTCGTCTAGGGATTCGGAAAGCTAA

                          cov    pid 401           .         .         .         .         :         .         .         . 480
1 EU104646_An.gambiae 100.0% 100.0%     GTCGCTGTAACTCATGTGGGCCCATACACGGCGTTGCGCTACCACGCTAAGTTAGCCCTACATAYACAAGCATCAACCCA
2 MT408564            100.0%  99.8%     GTCGCTGTAACTCATGTGGGCCCATACACGGCGTTGCGCTACCACGCTAAGTTAGCCCTACATATACAAGCATCAACCCA

                          cov    pid 481           .         5         .         .         .         .         :         . 560
1 EU104646_An.gambiae 100.0% 100.0%     CGGCACGGGCGTAGCTGTAATACTTACGTCTCGGTTATACCACGTAGGCCTCAAGTGATGTGTGACTACCCCCTAAATTT
2 MT408564            100.0%  99.8%     CGGCACGGGCGTAGCTGTAATACTTACGTCTCGGTTATACCACGTAGGCCTCAAGTGATGTGTGACTACCCCCTAAATTT

                          cov    pid 561      ] 566
1 EU104646_An.gambiae 100.0% 100.0%     AAGCAT
2 MT408564            100.0%  99.8%     AAGCAT
```

(c)

```
Reference sequence (1): MK043038_Anopheles
Identities normalised by aligned length.
Colored by: identity

                          cov    pid   1 [         .         .         .         .         :         .         .         . 80
1 MK043038_Anopheles 100.0% 100.0%     --------GCAGGACACATGAACACCGACACGTTGAACGCATATGGCGCATCGGACGTTTAAACCCGACCGATGCTCACA
2 MT408582           100.0%  98.5%     TGTGAACTGCAGGACACATGAACACCGACACGTTGAACGCATATGGCGCATCGGACGTTTAAACCCGACCGATGCTCACA

                          cov    pid  81           .         1         .         .         .         .         :         . 160
1 MK043038_Anopheles 100.0% 100.0%     TTCTTGAGTGCCTACCAAATCCTTGTTACACAAATACCTGACTACAGAGCGCGCGTGATGCGACGACCAAACACTACCGG
2 MT408582           100.0%  98.5%     TTCTTGAGTGCCTACCAAATCCTTGTTACACAAATACCTGACTACAGAGCGCGCGTGATGCGACGACCAAACACTACCGG

                          cov    pid 161           .         .         .         2         .         .         .         . 240
1 MK043038_Anopheles 100.0% 100.0%     CGCACCAACCGCCGTAGTGTGTCGCTGGCTGGACACGCGAGCGCACTGTGCATAATGGCGTGCTCGGCTCCCACCTGCGG
2 MT408582           100.0%  98.5%     CGCACCAACCGCCGTAGTGTGTCGCTGGCTGGACACGCGAGCGCACTGTGCATAATGGCGTGCTCGGCTCCCACCTGCGG

                          cov    pid 241           :         .         .         .         .         3         .         . 320
1 MK043038_Anopheles 100.0% 100.0%     GTGGGGAGCGCGGGGGCGCTGAAAGGTAAGGCTATACACTGTGACCTCAAAGGTGCGGGTCAAGTCGTGCGGATCGACCG
2 MT408582           100.0%  98.5%     GTGGGGAGCGCGGGGGCGCTGAAAGGTAAGGCTATACACTGTGACCTCAAAGGTGCGGGTCAAGTCGTGCGGATCGACCG

                          cov    pid 321           .         .         :         .         .         .         .         4 400
1 MK043038_Anopheles 100.0% 100.0%     TCAGGCTACATGACGACCTGAGAGCCCCGGCTGCCTAACTCACAGTAGGCGACGCCGTCCGTTCTCAGGGTCCATCGTCT
2 MT408582           100.0%  98.5%     TCAGGCTACATGACGACCTGAGAGCCCCGGCTGCCTAACTCACAGTAGGCGACGCCGTCCGTTCTCAGGGTCCATCGTCT

                          cov    pid 401           .         .         .         .         :         .         .         . 480
1 MK043038_Anopheles 100.0% 100.0%     GGCCGGGTCGTGTAACGCGTGCGACCCATACCCGGGCCTACTTATGTGCAGTGTACGTTTGCCTCCTTTCACCATATCAG
2 MT408582           100.0%  98.5%     GGCCGGGTCGTGTAACGCGTGCGACCCATACCCGGGCCTACTTATGTGCAGTGTACGTTTGCCTCCTTTCACCATATCAG

                          cov    pid 481           .         5         .         .         .  ] 533
1 MK043038_Anopheles 100.0% 100.0%     GACAACCAAGTAGGCCTCAAGTGATGTGCGACAACCCCCTGAATTTAAGCATA
2 MT408582           100.0%  98.5%     GACAACCAAGTAGGCCTCAAGTGATGTGCGACAACCCCCTGAATTTAAGCATA
```

(d)

```
Reference sequence (1): An.funestus_NC038158
Identities normalised by aligned length.
Colored by: identity
                              cov    pid   1 [         .         .         .         .         :         .         .         . 80
1 An.funestus_NC038158 100.0% 100.0%     GCTGGAATAGTAGGAACTTCTTTAAGAATCCTTATTCGAGCCGAATTAGGACATCCCGGAGCATTTATTGGAGACGATCA
2 An.funestus_H01      100.0%  99.5%     GCTGGAATAGTAGGAACTTCTTTAAGAATCCTTATTCGAGCCGAATTAGGACATCCCGGAGCATTTATTGGAGACGATCA
3 An.funestus_H02      100.0%  99.7%     GCTGGAATAGTAGGAACTTCTTTAAGAATCCTTATTCGAGCCGAATTAGGACATCCCGGAGCATTTATTGGAGACGATCA
4 An.funestus_H03      100.0%  99.4%     GCTGGAATAGTAGGAACTTCTTTAAGAATCCTTATTCGAGCCGAATTAGGACATCCCGGAGCATTTATTGGAGACGATCA
5 An.funestus_H04      100.0%  99.4%     GCTGGAATAGTAGGAACTTCTTTAAGAATCCTTATTCGAGCCGAATTAGGACATCCCGGAGCATTTATTGGAGACGATCA
6 An.funestus_H05      100.0%  99.4%     GCTGGAATAGTAGGAACTTCTTTAAGAATCCTTATTCGAGCCGAATTAGGACATCCCGGAGCATTTATTGGAGACGATCA
7 An.funestus_H06      100.0%  99.2%     GCTGGAATAGTAGGAACTTCTTTAAGAATCCTTATTCGAGCCGAATTAGGACATCCCGGAGCATTTATTGGAGACGATCA

                              cov    pid  81           .         1         .         .         .         .         :         . 160
1 An.funestus_NC038158 100.0% 100.0%     AATTTATAATGTAATTGTTACTGCTCATGCTTTTATTATAATTTTTTTTATAGTAATACCTATTATAATTGGAGGATTTG
2 An.funestus_H01      100.0%  99.5%     AATTTATAATGTAATTGTTACTGCTCATGCTTTTATTATAATTTTTTTTATAGTAATACCTATTATAATTGGAGGATTTG
3 An.funestus_H02      100.0%  99.7%     AATTTATAATGTAATTGTTACTGCTCATGCTTTTATTATAATTTTTTTTATAGTAATACCTATTATAATTGGAGGATTTG
4 An.funestus_H03      100.0%  99.4%     AATTTATAATGTAATTGTTACTGCTCATGCTTTTATTATAATTTTTTTTATAGTAATACCTATTATAATTGGAGGATTTG
5 An.funestus_H04      100.0%  99.4%     AATTTATAATGTAATTGTTACTGCTCATGCTTTTATTATAATTTTTTTTATAGTAATACCTATTATAATTGGAGGATTTG
6 An.funestus_H05      100.0%  99.4%     AATTTATAATGTAATTGTTACTGCTCATGCTTTTATTATAATTTTTTTTATAGTAATACCTATTATAATTGGAGGATTTG
7 An.funestus_H06      100.0%  99.2%     AATTTATAATGTAATTGTTACTGCTCATGCTTTTATTATAATTTTTTTTATAGTAATACCTATTATAATTGGGGGATTTG

                              cov    pid 161           .         .         .         2         .         .         .         . 240
1 An.funestus_NC038158 100.0% 100.0%     GAAATTGACTTGTACCTTTAATATTAGGAGCCCCTGATATAGCTTTCCCTCGAATAAATAATATAAGATTTTGAATACTT
2 An.funestus_H01      100.0%  99.5%     GAAATTGACTTGTACCTTTAATATTAGGAGCCCCTGATATAGCTTTCCCTCGAATAAATAATATAAGATTTTGAATACTT
3 An.funestus_H02      100.0%  99.7%     GAAATTGACTTGTACCTTTAATATTAGGAGCCCCTGATATAGCTTTCCCTCGAATAAATAATATAAGATTTTGAATACTT
4 An.funestus_H03      100.0%  99.4%     GAAATTGACTTGTACCTTTAATATTAGGAGCCCCTGATATAGCTTTCCCTCGAATAAATAATATAAGATTTTGAATACTT
5 An.funestus_H04      100.0%  99.4%     GAAATTGACTTGTACCTTTAATATTAGGAGCCCCTGATATAGCTTTCCCTCGAATAAATAATATAAGATTTTGAATACTT
6 An.funestus_H05      100.0%  99.4%     GAAATTGACTTGTACCTTTAATATTAGGAGCCCCTGATATAGCTTTCCCTCGAATAAATAATATAAGATTTTGAATACTT
7 An.funestus_H06      100.0%  99.2%     GAAATTGACTTGTACCTTTAATATTAGGAGCCCCTGATATAGCTTTCCCTCGAATAAATAATATAAGATTTTGAATACTT

                              cov    pid 241           :         .         .         .         .         3         .         . 320
1 An.funestus_NC038158 100.0% 100.0%     CCTCCTTCATTAACTTTACTTCTTTCTAGTAGTATAGTAGAAAATGGAGCAGGAACAGGATGAACAGTTTATCCCCCTTT
2 An.funestus_H01      100.0%  99.5%     CCTCCTTCATTAACTTTACTTCTTTCTAGTAGTATAGTAGAAAATGGAGCAGGAACAGGATGAACAGTTTATCCCCCTTT
3 An.funestus_H02      100.0%  99.7%     CCTCCTTCATTAACTTTACTTCTTTCTAGTAGTATAGTAGAAAATGGAGCAGGAACAGGATGAACAGTTTATCCCCCTTT
4 An.funestus_H03      100.0%  99.4%     CCTCCTTCATTAACTTTACTTCTTTCTAGTAGTATAGTAGAAAATGGAGCAGGAACAGGATGAACAGTTTATCCCCCTTT
5 An.funestus_H04      100.0%  99.4%     CCTCCTTCATTAACTTTACTTCTTTCTAGTAGTATAGTAGAAAATGGAGCAGGAACAGGATGAACAGTTTATCCCCCTTT
6 An.funestus_H05      100.0%  99.4%     CCTCCTTCATTAACTTTACTTCTTTCTAGTAGTATAGTAGAAAATGGAGCAGGAACAGGATGAACAGTTTATCCTCCTTT
7 An.funestus_H06      100.0%  99.2%     CCTCCTTCATTAACTTTACTTCTTTCTAGTAGTATAGTAGAAAATGGAGCAGGAACAGGATGAACAGTTTATCCCCCTTT

                              cov    pid 321           .         .         :         .         .         .         .         4 400
1 An.funestus_NC038158 100.0% 100.0%     ATCATCAGGAATTGCTCATGCTGGTGCTTCAGTTGATTTAGCTATTTTTTCATTACATTTAGCAGGAATTTCTTCTATTT
2 An.funestus_H01      100.0%  99.5%     ATCATCAGGAATTGCTCATGCTGGTGCTTCAGTTGATTTAGCTATTTTTTCATTACATTTAGCAGGAATTTCTTCTATTT
3 An.funestus_H02      100.0%  99.7%     ATCATCAGGAATTGCTCATGCTGGTGCTTCAGTTGATTTAGCTATTTTTTCATTACATTTAGCAGGAATTTCTTCTATTT
4 An.funestus_H03      100.0%  99.4%     ATCATCAGGAATTGCTCATGCTGGTGCTTCAGTTGATTTAGCTATTTTTTCATTACATTTAGCAGGAATTTCTTCTATTT
5 An.funestus_H04      100.0%  99.4%     ATCATCAGGAATTGCTCATGCTGGTGCTTCAGTTGATTTAGCTATTTTTTCATTACATTTAGCAGGAATTTCTTCTATTT
6 An.funestus_H05      100.0%  99.4%     ATCATCAGGAATTGCTCATGCTGGTGCTTCAGTTGATTTAGCTATTTTTTCATTACATTTAGCAGGAATTTCTTCTATTT
7 An.funestus_H06      100.0%  99.2%     ATCATCAGGAATTGCTCATGCTGGTGCTTCAGTTGATTTAGCTATTTTTTCATTACATTTAGCAGGAATTTCTTCTATTT

                              cov    pid 401           .         .         .         .         :         .         .         . 480
1 An.funestus_NC038158 100.0% 100.0%     TAGGAGCAGTAAATTTTATTACAACTGTAATTAATATACGATCTCCTGGAATTACTTTAGATCGAATACCATTATTTGTC
2 An.funestus_H01      100.0%  99.5%     TAGGAGCAGTAAATTTTATTACAACTGTAATTAATATACGATCTCCTGGAATTACTTTAGATCGAATACCATTATTTGTC
3 An.funestus_H02      100.0%  99.7%     TAGGAGCAGTAAATTTTATTACAACTGTAATTAATATACGATCTCCTGGAATTACTTTAGATCGAATACCATTATTTGTC
4 An.funestus_H03      100.0%  99.4%     TAGGAGCAGTAAATTTTATTACAACTGTAATTAATATACGATCTCCTGGAATTACTTTAGACCGAATACCATTATTTGTC
5 An.funestus_H04      100.0%  99.4%     TAGGAGCAGTAAATTTTATTACAACTGTAATTAATATACGATCTCCTGGAATTACTTTAGATCGAATACCATTATTTGTC
6 An.funestus_H05      100.0%  99.4%     TAGGAGCAGTAAATTTTATTACAACTGTAATTAATATACGATCTCCTGGAATTACTTTAGATCGAATACCATTATTTGTC
7 An.funestus_H06      100.0%  99.2%     TAGGAGCAGTAAATTTTATTACAACTGTAATTAATATACGATCTCCTGGAATTACTTTAGATCGAATACCATTATTTGTC

                              cov    pid 481           .         5         .         .         .         .         :         . 560
1 An.funestus_NC038158 100.0% 100.0%     TGATCTGTAGTAATTACAGCAATTCTTTTATTGCTTTCTTTACCAGTATTAGCTGGAGCTATTACAATACTATTAACAGA
2 An.funestus_H01      100.0%  99.5%     TGATCTGTAGTAATTACAGCAATTCTTTTATTACTTTCTTTACCAGTATTAGCTGGAGCTATTACAATATTATTAACAGA
3 An.funestus_H02      100.0%  99.7%     TGATCTGTAGTAATTACAGCAATTCTTTTATTACTTTCTTTACCAGTATTAGCTGGAGCTATTACAATATTATTAACAGA
4 An.funestus_H03      100.0%  99.4%     TGATCTGTAGTAATTACAGCAATTCTTTTATTACTTTCTTTACCAGTATTAGCTGGAGCTATTACAATATTATTAACAGA
5 An.funestus_H04      100.0%  99.4%     TGATCTGTAGTAATTACAGCAATTCTTTTATTACTTTCTTTACCAGTATTAGCTGGGGCTATTACAATATTATTAACAGA
6 An.funestus_H05      100.0%  99.4%     TGATCTGTAGTAATTACAGCAATTCTTTTATTACTTTCTTTACCAGTATTAGCTGGAGCTATTACAATATTATTAACAGA
7 An.funestus_H06      100.0%  99.2%     TGATCTGTAGTAATTACAGCAATTCTTTTATTACTTTCTTTACCAGTATTAGCTGGGGCTATTACAATATTATTAACAGA

                              cov    pid 561           .         .         .         6         .         .         ] 636
1 An.funestus_NC038158 100.0% 100.0%     TCGAAATTTAAATACTTCTTTTTTTGATCCTGCAGGAGGAGGAGATCCAATTTTATACCAACACTTATTTTGATTT
2 An.funestus_H01      100.0%  99.5%     TCGAAATTTAAATACTTCTTTTTTTGATCCTGCAGGAGGAGGAGATCCAATTTTATATCAACACTTATTTTGATTT
3 An.funestus_H02      100.0%  99.7%     TCGAAATTTAAATACTTCTTTTTTTGATCCTGCAGGAGGAGGAGATCCAATTTTATACCAACACTTATTTTGATTT
4 An.funestus_H03      100.0%  99.4%     TCGAAATTTAAATACTTCTTTTTTTGATCCTGCAGGAGGAGGAGATCCAATTTTATATCAACACTTATTTTGATTT
5 An.funestus_H04      100.0%  99.4%     TCGAAATTTAAATACTTCTTTTTTTGATCCTGCAGGAGGAGGAGATCCAATTTTATATCAACACTTATTTTGATTT
6 An.funestus_H05      100.0%  99.4%     TCGAAATTTAAATACTTCTTTTTTTGATCCTGCAGGAGGAGGAGATCCAATTTTATATCAACACTTATTTTGATTT
7 An.funestus_H06      100.0%  99.2%     TCGAAATTTAAATACTTCTTTTTTTGATCCTGCAGGAGGAGGAGATCCAATTTTATATCAACACTTATTTTGATTT
```

(e)

```
Reference sequence (1): An.gambiae_NC002084
Identities normalised by aligned length.
Colored by: identity

                           cov    pid   1 [         .         .         .         .         :         .         .         . 80
1 An.gambiae_NC002084 100.0% 100.0%     GCTGGAATAGTAGGGACATCTTTAAGAATCCTAATTCGAGCTGAACTAGGACACCCTGGAGCATTTATTGGAGACGATCA
2 An.gambiae_H01      100.0%  99.4%     GCTGGAATAGTAGGGACATCTTTAAGAATCCTAATTCGAGCTGAACTAGGACACCCTGGAGCATTTATTGGAGACGATCA
3 An.gambiae_H02      100.0% 100.0%     GCTGGAATAGTAGGGACATCTTTAAGAATCCTAATTCGAGCTGAACTAGGACACCCTGGAGCATTTATTGGAGACGATCA
4 An.gambiae_H03      100.0%  99.8%     GCTGGAATAGTAGGGACATCTTTAAGAATCCTAATTCGAGCTGAACTAGGACACCCTGGAGCATTTATTGGAGACGATCA
5 An.gambiae_H04      100.0%  99.8%     GCTGGAATAGTAGGGACATCTTTAAGAATCCTAATTCGAGCTGAACTAGGACACCCTGGAGCATTTATTGGAGACGATCA

                           cov    pid  81           .         1         .         .         .         .         :         . 160
1 An.gambiae_NC002084 100.0% 100.0%     AATTTATAATGTAATCGTTACTGCTCATGCTTTTATTATAATTTTCTTTATAGTTATACCTATTATAATTGGAGGGTTTG
2 An.gambiae_H01      100.0%  99.4%     AATTTATAATGTAATCGTTACTGCTCATGCTTTTATTATAATTTTCTTTATAGTTATACCTATTATAATTGGAGGATTTG
3 An.gambiae_H02      100.0% 100.0%     AATTTATAATGTAATCGTTACTGCTCATGCTTTTATTATAATTTTCTTTATAGTTATACCTATTATAATTGGAGGGTTTG
4 An.gambiae_H03      100.0%  99.8%     AATTTATAATGTAATCGTTACTGCTCATGCTTTTATTATAATTTTCTTTATAGTTATACCTATTATAATTGGAGGGTTTG
5 An.gambiae_H04      100.0%  99.8%     AATTTATAATGTAATCGTTACTGCTCATGCTTTTATTATAATTTTCTTTATAGTTATACCTATTATAATTGGAGGATTTG

                           cov    pid 161           .         .         .         2         .         .         .         . 240
1 An.gambiae_NC002084 100.0% 100.0%     GAAACTGATTAGTTCCTTTAATATTAGGAGCACCTGATATAGCTTTTCCTCGAATAAATAATATAAGATTTTGAATGCTT
2 An.gambiae_H01      100.0%  99.4%     GAAACTGATTAGTTCCTTTAATATTAGGAGCACCTGATATAGCTTTTCCTCGAATAAATAATATAAGATTTTGAATACTT
3 An.gambiae_H02      100.0% 100.0%     GAAACTGATTAGTTCCTTTAATATTAGGAGCACCTGATATAGCTTTTCCTCGAATAAATAATATAAGATTTTGAATGCTT
4 An.gambiae_H03      100.0%  99.8%     GAAACTGATTAGTTCCTTTAATATTAGGAGCACCTGATATAGCTTTTCCTCGAATAAATAATATAAGATTTTGAATGCTT
5 An.gambiae_H04      100.0%  99.8%     GAAACTGATTAGTTCCTTTAATATTAGGAGCACCTGATATAGCTTTTCCTCGAATAAATAATATAAGATTTTGAATGCTT

                           cov    pid 241           :         .         .         .         .         3         .         . 320
1 An.gambiae_NC002084 100.0% 100.0%     CCTCCTTCATTAACACTTTTAATTTCTAGTAGTATAGTAGAAAACGGGGCTGGAACAGGATGAACTGTTTATCCTCCTCT
2 An.gambiae_H01      100.0%  99.4%     CCTCCTTCATTAACACTTTTAATTTCTAGTAGTATAGTAGAAAACGGGGCTGGAACAGGATGAACTGTTTATCCTCCTCT
3 An.gambiae_H02      100.0% 100.0%     CCTCCTTCATTAACACTTTTAATTTCTAGTAGTATAGTAGAAAACGGGGCTGGAACAGGATGAACTGTTTATCCTCCTCT
4 An.gambiae_H03      100.0%  99.8%     CCTCCTTCATTAACACTTTTAATTTCTAGTAGTATAGTAGAAAACGGGGCTGGAACAGGATGAACTGTTTATCCTCCTCT
5 An.gambiae_H04      100.0%  99.8%     CCTCCTTCATTAACACTTTTAATTTCTAGTAGTATAGTAGAAAACGGGGCTGGAACAGGATGAACTGTTTATCCTCCTCT

                           cov    pid 321           .         .         :         .         .         .         .         4 400
1 An.gambiae_NC002084 100.0% 100.0%     ATCTTCTGGAATTGCTCATGCTGGAGCTTCAGTAGATTTAGCAATTTTTTCTCTTCATTTAGCAGGAATTTCTTCTATTT
2 An.gambiae_H01      100.0%  99.4%     ATCTTCTGGAATTGCTCATGCTGGAGCTTCAGTAGATTTAGCAATTTTTTCTCTTCATTTAGCAGGAATTTCTTCTATTT
3 An.gambiae_H02      100.0% 100.0%     ATCTTCTGGAATTGCTCATGCTGGAGCTTCAGTAGATTTAGCAATTTTTTCTCTTCATTTAGCAGGAATTTCTTCTATTT
4 An.gambiae_H03      100.0%  99.8%     ATCTTCTGGAATTGCTCATGCTGGAGCTTCAGTAGATTTAGCAATTTTTTCTCTTCATTTAGCAGGAATTTCTTCTATTT
5 An.gambiae_H04      100.0%  99.8%     ATCTTCTGGAATTGCTCATGCTGGAGCTTCAGTAGATTTAGCAATTTTTTCTCTTCATTTAGCAGGAATTTCTTCTATTT

                           cov    pid 401           .         .         .         .         :         .         .         . 480
1 An.gambiae_NC002084 100.0% 100.0%     TAGGAGCAGTAAATTTTATTACAACAGTAATTAATATACGGTCTCCAGGAATTACATTAGATCGAATACCATTATTTGTT
2 An.gambiae_H01      100.0%  99.4%     TAGGAGCAGTAAATTTTATTACAACAGTAATTAATATACGATCTCCAGGAATTACATTAGATCGAATACCATTATTTGTT
3 An.gambiae_H02      100.0% 100.0%     TAGGAGCAGTAAATTTTATTACAACAGTAATTAATATACGGTCTCCAGGAATTACATTAGATCGAATACCATTATTTGTT
4 An.gambiae_H03      100.0%  99.8%     TAGGAGCAGTAAATTTTATTACAACAGTAATTAATATACGGTCTCCAGGAATTACATTAGATCGAATACCATTATTTGTT
5 An.gambiae_H04      100.0%  99.8%     TAGGAGCAGTAAATTTTATTACAACAGTAATTAATATACGGTCTCCAGGAATTACATTAGATCGAATACCATTATTTGTT

                           cov    pid 481           .         5         .         .         .         .         :         . 560
1 An.gambiae_NC002084 100.0% 100.0%     TGATCGGTAGTTATTACAGCAGTATTATTATTATTATCATTACCAGTATTAGCAGGAGCTATTACTATATTATTAACTGA
2 An.gambiae_H01      100.0%  99.4%     TGATCGGTAGTTATTACAGCAGTATTATTATTATTATCATTACCAGTATTAGCAGGAGCTATTACTATATTATTAACTGA
3 An.gambiae_H02      100.0% 100.0%     TGATCGGTAGTTATTACAGCAGTATTATTATTATTATCATTACCAGTATTAGCAGGAGCTATTACTATATTATTAACTGA
4 An.gambiae_H03      100.0%  99.8%     TGATCGGTAGTTATTACAGCAGTATTATTATTATTATCATTACCAGTATTAGCAGGAGCTATTACTATATTATTAACTGA
5 An.gambiae_H04      100.0%  99.8%     TGATCGGTAGTTATTACAGCAGTATTATTATTATTATCATTACCAGTATTAGCAGGAGCTATTACTATATTATTAACTGA

                           cov    pid 561           .         .         .         6         .         .         .       ] 636
1 An.gambiae_NC002084 100.0% 100.0%     TCGAAATTTAAATACATCTTTCTTTGATCCAGCAGGAGGAGGTGATCCAATTTTATATCAACACTTATTCTGATTT
2 An.gambiae_H01      100.0%  99.4%     TCGAAATTTAAATACATCTTTCTTTGATCCAGCAGGAGGGGGTGATCCAATTTTATATCAACACTTATTCTGATTT
3 An.gambiae_H02      100.0% 100.0%     TCGAAATTTAAATACATCTTTCTTTGATCCAGCAGGAGGAGGTGATCCAATTTTATATCAACACTTATTCTGATTT
4 An.gambiae_H03      100.0%  99.8%     TCGAAATTTAAATACATCTTTCTTTGACCCAGCAGGAGGAGGTGATCCAATTTTATATCAACACTTATTCTGATTT
5 An.gambiae_H04      100.0%  99.8%     TCGAAATTTAAATACATCTTTCTTTGATCCAGCAGGAGGAGGTGATCCAATTTTATATCAACACTTATTCTGATTT
```

(f)

```
Reference sequence (1): An.sp.D_MK047667
Identities normalised by aligned length.
Colored by: identity

                       cov    pid   1 [         .         .         .         :         .         .         . 80
 1 An.sp.D_MK047667 100.0% 100.0%     GCAGGAATAGTAGGAACATCATTAAGAATTCTTATTCGAGCAGAATTAGGACATCCAGGAGCATTTATTGGGGATGATCA
 2 An.sp.F_MK047669 100.0%  98.9%     GCAGGAATAGTAGGAACATCATTAAGAATTCTTATTCGAGCAGAATTAGGACATCCAGGAGCATTTATTGGGGATGATCA
 3 An.sp.C_MK047666 100.0%  99.1%     GCAGGAATAGTAGGAACATCATTAAGAATTCTTATTCGAGCAGAATTAGGACATCCAGGAGCATTTATTGGGGATGATCA
 4 An.sp.17_H01     100.0%  98.6%     GCAGGAATAGTAGGAACATCATTAAGAATTCTTATTCGAGCAGAATTAGGACATCCAGGAGCATTTATTGGGGATGATCA
 5 An.sp.17_H02     100.0%  99.2%     GCAGGAATAGTAGGAACATCATTAAGAATTCTTATTCGAGCAGAATTAGGACATCCAGGAGCATTTATTGGGGATGATCA
 6 An.sp.17_H03     100.0%  99.4%     GCAGGAATAGTAGGAACATCATTAAGAATTCTTATTCGAGCAGAATTAGGACATCCAGGAGCATTTATTGGGGATGATCA
 7 An.sp.17_H04     100.0%  98.6%     GCAGGAATAGTAGGAACATCATTAAGAATTCTTATTCGAACAGAATTAGGACATCCAGGAGCATTTATTGGGGATGATCA
 8 An.sp.17_H05     100.0%  99.8%     GCAGGAATAGTAGGAACATCATTAAGAATTCTTATTCGAGCAGAATTAGGACATCCAGGAGCATTTATTGGGGATGATCA
 9 An.sp.17_H06     100.0%  99.1%     GCAGGAATAGTAGGAACATCATTAAGAATTCTTATTCGAGCAGAATTAGGACATCCAGGAGCATTTATTGGGGATGATCA
10 An.sp.17_H07     100.0%  99.4%     GCAGGAATAGTAGGAACATCATTAAGAATTCTTATTCGAGCAGAATTAGGACATCCAGGAGCATTTATTGGGGATGATCA
11 An.sp.17_H08     100.0%  99.7%     GCAGGAATAGTAGGAACATCATTAAGAATTCTTATTCGAGCAGAATTAGGACATCCAGGAGCATTTATTGGGGATGATCA
12 An.sp.17_H09     100.0%  99.5%     GCAGGAATAGTAGGAACATCATTAAGAATTCTTATTCGAGCAGAATTAGGACATCCAGGAGCATTTATTGGGGATGATCA
13 An.sp.17_H10     100.0%  99.5%     GCAGGAATAGTAGGAACATCATTAAGAATTCTTATTCGAGCAGAATTAGGACATCCAGGAGCATTTATTGGGGATGATCA
14 An.sp.17_H11     100.0%  98.9%     GCAGGAATAGTAGGAACATCATTAAGAATTCTTATTCGAGCAGAATTAGGACATCCAGGAGCATTTATTGGGGATGATCA
15 An.sp.17_H12     100.0%  99.5%     GCAGGAATAGTAGGAACATCATTAAGAATTCTTATTCGAGCAGAATTAGGACATCCAGGAGCATTTATTGGGGATGATCA
16 An.sp.17_H13     100.0%  98.9%     GCAGGAATAGTAGGAACATCATTAAGAATTCTTATTCGAGCAGAATTAGGACATCCAGGAGCATTTATTGGGGATGATCA

                       cov    pid  81           .         1         .         .         .         .         :         . 160
 1 An.sp.D_MK047667 100.0% 100.0%     AATTTATAATGTAATTGTTACTGCTCATGCTTTTATTATAATTTTCTTTATAGTTATACCTATTATAATTGGAGGGTTTG
 2 An.sp.F_MK047669 100.0%  98.9%     AATTTATAATGTAATTGTTACTGCTCATGCTTTTATTATAATTTTCTTTATAGTTATACCTATTATAATTGGAGGGTTTG
 3 An.sp.C_MK047666 100.0%  99.1%     AATTTATAATGTAATTGTTACTGCTCATGCTTTTATTATAATTTTCTTTATAGTTATACCTATTATAATTGGAGGGTTTG
 4 An.sp.17_H01     100.0%  98.6%     AATTTATAATGTAATTGTTACTGCTCATGCTTTTATTATAATTTTCTTTATAGTTATACCTATTATAATTGGAGGGTTTG
 5 An.sp.17_H02     100.0%  99.2%     AATTTATAATGTAATTGTTACTGCTCATGCTTTTATTATAATTTTCTTTATAGTTATACCTATTATAATTGGAGGGTTTG
 6 An.sp.17_H03     100.0%  99.4%     AATTTATAATGTAATTGTTACTGCTCATGCTTTTATTATAATTTTCTTTATAGTTATACCTATTATAATTGGAGGGTTTG
 7 An.sp.17_H04     100.0%  98.6%     AATTTATAATGTAATTGTTACTGCTCATGCTTTTATTATAATTTTCTTTATAGTTATACCTATTATAATTGGAGGGTTTG
 8 An.sp.17_H05     100.0%  99.8%     AATTTATAATGTAATTGTTACTGCTCATGCTTTTATTATAATTTTCTTTATAGTTATACCTATTATAATTGGAGGGTTTG
 9 An.sp.17_H06     100.0%  99.1%     AATTTATAATGTAATTGTTACTGCTCATGCTTTTATTATAATTTTCTTTATAGTTATACCTATTATAATTGGAGGGTTTG
10 An.sp.17_H07     100.0%  99.4%     AATTTATAATGTAATTGTTACTGCTCATGCTTTTATTATAATTTTCTTTATAGTTATACCTATTATAATTGGAGGGTTTG
11 An.sp.17_H08     100.0%  99.7%     AATTTATAATGTAATTGTTACTGCTCATGCTTTTATTATAATTTTCTTTATAGTTATACCTATTATAATTGGAGGGTTTG
12 An.sp.17_H09     100.0%  99.5%     AATTTATAATGTAATTGTTACTGCTCATGCTTTTATTATAATTTTCTTTATAGTTATACCTATTATAATTGGAGGGTTTG
13 An.sp.17_H10     100.0%  99.5%     AATTTATAATGTAATTGTTACTGCTCATGCTTTTATTATAATTTTCTTTATAGTTATACCTATTATAATTGGAGGGTTTG
14 An.sp.17_H11     100.0%  98.9%     AATTTATAATGTAATTGTTACTGCTCATGCTTTTATTATAATTTTCTTTATAGTTATACCTATTATAATTGGAGGGTTTG
15 An.sp.17_H12     100.0%  99.5%     AATTTATAATGTAATTGTTACTGCTCATGCTTTTATTATAATTTTCTTTATAGTTATACCTATTATAATTGGAGGGTTTG
16 An.sp.17_H13     100.0%  98.9%     AATTTATAATGTAATTGTTACTGCTCATGCTTTTATTATAATTTTCTTTATAGTTATACCTATTATAATTGGAGGGTTTG

                       cov    pid 161           .         .         .         2         .         .         .         . 240
 1 An.sp.D_MK047667 100.0% 100.0%     GAAATTGACTTGTTCCTTTAATACTAGGAGCTCCAGATATGGCATTTCCTCGAATAAATAATATAAGATTTTGAATATTA
 2 An.sp.F_MK047669 100.0%  98.9%     GAAATTGACTTGTTCCTTTAATACTAGGAGCTCCAGATATAGCGTTCCCTCGAATAAATAATATAAGATTTTGAATATTA
 3 An.sp.C_MK047666 100.0%  99.1%     GAAATTGACTTGTTCCTTTAATACTAGGAGCCCCAGATATGGCATTTCCTCGAATAAATAATATAAGATTTTGAATATTA
 4 An.sp.17_H01     100.0%  98.6%     GAAATTGACTTGTTCCTTTAATACTAGGAGCTCCAGATATAGCATTTCCTCGAATAAATAATATAAGATTTTGAATATTA
 5 An.sp.17_H02     100.0%  99.2%     GAAATTGACTTGTTCCTTTAATACTAGGAGCTCCAGATATGGCATTTCCTCGAATAAATAATATAAGATTTTGAATATTA
 6 An.sp.17_H03     100.0%  99.4%     GAAATTGACTTGTTCCTTTAATACTAGGAGCCCCAGATATGGCATTTCCTCGAATAAATAATATAAGATTTTGAATATTA
 7 An.sp.17_H04     100.0%  98.6%     GAAATTGACTTGTTCCTTTAATACTAGGAGCCCCAGATATGGCATTTCCTCGAATAAATAATATAAGATTTTGAATATTA
 8 An.sp.17_H05     100.0%  99.8%     GAAATTGACTTGTTCCTTTAATACTAGGAGCTCCAGATATGGCATTTCCTCGAATAAATAATATAAGATTTTGAATATTA
 9 An.sp.17_H06     100.0%  99.1%     GAAATTGACTTGTTCCTTTAATACTAGGAGCTCCAGATATGGCATTTCCTCGAATAAATAATATAAGATTTTGAATATTA
10 An.sp.17_H07     100.0%  99.4%     GAAATTGACTTGTTCCTTTAATACTAGGAGCTCCAGATATGGCATTTCCTCGAATAAATAATATAAGATTTTGAATATTA
11 An.sp.17_H08     100.0%  99.7%     GAAATTGACTTGTTCCTTTAATACTAGGAGCCCCAGATATGGCATTTCCTCGAATAAATAATATAAGATTTTGAATATTA
12 An.sp.17_H09     100.0%  99.5%     GAAATTGACTTGTTCCTTTAATACTAGGAGCTCCAGATATAGCATTTCCTCGAATAAATAATATAAGATTTTGAATATTA
13 An.sp.17_H10     100.0%  99.5%     GAAATTGACTTGTTCCTTTAATACTAGGAGCCCCAGATATGGCATTTCCTCGAATAAATAATATAAGATTTTGAATATTA
14 An.sp.17_H11     100.0%  98.9%     GAAATTGACTTGTTCCTTTAATACTAGGAGCTCCAGATATAGCGTTCCCTCGAATAAATAATATAAGATTTTGAATATTA
15 An.sp.17_H12     100.0%  99.5%     GAAATTGACTTGTTCCTTTAATACTAGGAGCTCCAGATATGGCATTTCCTCGAATAAATAATATAAGATTTTGAATATTA
16 An.sp.17_H13     100.0%  98.9%     GAAATTGACTTGTTCCTTTAATACTAGGAGCTCCAGATATAGCGTTCCCTCGAATAAATAATATAAGATTTTGAATATTA

                       cov    pid 241           :         .         .         .         .         3         .         . 320
 1 An.sp.D_MK047667 100.0% 100.0%     CCCCCTTCTTTAACTCTTCTTATTTCTAGAAGTATAGTAGAAAATGGAGCAGGAACTGGATGAACAGTATACCCTCCTTT
 2 An.sp.F_MK047669 100.0%  98.9%     CCTCCTTCTTTAACTCTTCTTATTTCTAGAAGTATAGTAGAAAATGGAGCAGGAACTGGATGAACAGTATACCCTCCTTT
 3 An.sp.C_MK047666 100.0%  99.1%     CCCCCCTCTTTAACTCTTCTTATTTCTAGAAGTATAGTAGAAAATGGAGCAGGAACTGGATGAACAGTATACCCTCCTTT
 4 An.sp.17_H01     100.0%  98.6%     CCTCCTTCTTTAACTCTTCTTATTTCTAGTAGTATAGTAGAAAATGGAGCAGGAACTGGATGAACAGTATACCCTCCTTT
 5 An.sp.17_H02     100.0%  99.2%     CCTCCTTCTTTAACTCTTCTTATTTCTAGAAGTATAGTAGAAAATGGAGCAGGAACTGGATGAACAGTATACCCTCCTTT
 6 An.sp.17_H03     100.0%  99.4%     CCCCCCTCTTTAACTCTTCTTATTTCTAGAAGTATAGTAGAAAATGGAGCAGGAACTGGATGAACAGTATACCCTCCTTT
 7 An.sp.17_H04     100.0%  98.6%     CCTCCTTCTTTAACTCTTCTTATTTCTAGAAGTATAGTAGAAAATGGAGCAGGAACTGGATGAACAGTATACCCTCCTTT
 8 An.sp.17_H05     100.0%  99.8%     CCCCCTTCTTTAACTCTTCTTATTTCTAGAAGTATAGTAGAAAATGGAGCAGGAACTGGATGAACAGTATACCCTCCTTT
 9 An.sp.17_H06     100.0%  99.1%     CCTCCTTCTTTAACTCTTCTTATTTCTAGAAGTATAGTAGAAAATGGAGCAGGAACTGGATGAACAGTATACCCTCCTTT
10 An.sp.17_H07     100.0%  99.4%     CCCCCCTCTTTAACTCTTCTTATTTCTAGAAGTATAGTAGAAAATGGAGCAGGAACTGGATGAACAGTATACCCTCCTTT
11 An.sp.17_H08     100.0%  99.7%     CCCCCTTCTTTAACTCTTCTTATTTCTAGAAGTATAGTAGAAAATGGAGCAGGAACTGGATGAACAGTATACCCTCCTTT
12 An.sp.17_H09     100.0%  99.5%     CCCCCTTCTTTAACTCTTCTTATTTCTAGAAGTATAGTAGAAAATGGAGCAGGAACTGGATGAACAGTATACCCTCCTTT
13 An.sp.17_H10     100.0%  99.5%     CCCCCTTCTTTAACTCTTCTTATTTCTAGAAGTATAGTAGAAAATGGAGCAGGAACTGGATGAACAGTATACCCTCCTTT
14 An.sp.17_H11     100.0%  98.9%     CCTCCTTCTTTAACTCTTCTTATTTCTAGAAGTATAGTAGAAAATGGAGCAGGAACTGGATGAACAGTATACCCTCCTTT
15 An.sp.17_H12     100.0%  99.5%     CCCCCCTCTTTAACTCTTCTTATTTCTAGAAGTATAGTAGAAAATGGAGCAGGAACTGGATGAACAGTATACCCTCCTTT
16 An.sp.17_H13     100.0%  98.9%     CCTCCTTCTTTAACTCTTCTTATTTCTAGAAGTATAGTAGAAAATGGAGCAGGAACTGGATGAACAGTATACCCTCCTTT

                       cov    pid 321           .         .         :         .         .         .         .         4 400
 1 An.sp.D_MK047667 100.0% 100.0%     ATCTTCTGGAATTGCTCATGCAGGGGCTTCAGTTGATTTAGCTATTTTTTCTTTACATTTAGCTGGAATTTCTTCAATTT
```

```
 2 An.sp.F_MK047669 100.0%  98.9%   ATCTTCTGGAATTGCTCATGCAGGAGCTTCAGTTGATTTAGCTATTTTTTCTTTACATTTAGCTGGGATTTCTTCAATTT
 3 An.sp.C_MK047666 100.0%  99.1%   ATCTTCTGGAATTGCTCATGCAGGGGCTTCAGTTGATTTAGCTATTTTTTCTTTACACTTAGCTGGAATTTCTTCAATTT
 4 An.sp.17_H01     100.0%  98.6%   ATCTTCTGGGATTGCTCATGCAGGAGCTTCAGTTGATTTAGCTATTTTTTCTTTACATTTAGCTGGAATTTCTTCAATTT
 5 An.sp.17_H02     100.0%  99.2%   ATCTTCTGGGATTGCTCATGCAGGGGCTTCAGTTGATTTAGCTATTTTTTCTTTACATTTAGCTGGAATTTCTTCAATTT
 6 An.sp.17_H03     100.0%  99.4%   ATCTTCTGGAATTGCTCATGCAGGGGCTTCAGTTGATTTAGCTATTTTTTCTTTACATTTAGCTGGAATTTCTTCAATTT
 7 An.sp.17_H04     100.0%  98.6%   ATCTTCTGGGATTGCTCATGCGGGAGCTTCAGTTGATTTAGCTATTTTTTCTTTACATTTAGCTGGAATTTCTTCAATTT
 8 An.sp.17_H05     100.0%  99.8%   ATCTTCTGGAATTGCTCATGCAGGGGCTTCAGTTGATTTAGCTATTTTTTCTTTACATTTAGCTGGAATTTCTTCAATTT
 9 An.sp.17_H06     100.0%  99.1%   ATCTTCTGGGATTGCTCATGCAGGAGCTTCAGTTGATTTAGCTATTTTTTCTTTACATTTAGCTGGAATTTCTTCAATTT
10 An.sp.17_H07     100.0%  99.4%   ATCTTCTGGAATTGCTCATGCGGGGGCTTCAGTTGATTTAGCTATTTTTTCTTTACATTTAGCTGGAATTTCTTCAATTT
11 An.sp.17_H08     100.0%  99.7%   ATCTTCTGGAATTGCTCATGCAGGGGCTTCAGTTGATTTAGCTATTTTTTCTTTACATTTAGCTGGAATTTCTTCAATTT
12 An.sp.17_H09     100.0%  99.5%   ATCTTCTGGAATTGCTCATGCAGGGGCTTCAGTTGATTTAGCTATTTTTTCTTTACATTTAGCTGGAATTTCTTCAATTT
13 An.sp.17_H10     100.0%  99.5%   ATCTTCTGGAATTGCTCATGCAGGGGCTTCAGTTGATTTAGCTATTTTTTCTTTACATTTAGCTGGAATTTCTTCAATTT
14 An.sp.17_H11     100.0%  98.9%   ATCTTCTGGAATTGCTCATGCAGGGGCTTCAGTTGATTTAGCTATTTTTTCTTTACATTTAGCTGGAATTTCTTCAATTT
15 An.sp.17_H12     100.0%  99.5%   ATCTTCTGGAATTGCTCATGCAGGGGCTTCAGTTGATTTAGCTATTTTTTCTTTACATTTAGCTGGAATTTCTTCAATTT
16 An.sp.17_H13     100.0%  98.9%   ATCTTCTGGAATTGCTCATGCAGGAGCTTCAGTTGATTTAGCTATTTTTTCTTTACATTTAGCTGGAATTTCTTCAATTT

                     cov    pid 401          .         .         .         .         :         .         .         . 480
 1 An.sp.D_MK047667 100.0% 100.0%   TAGGGGCAGTAAATTTTATTACTACTGTCATTAATATACGATCTCCTGGAATTACATTAGATCGTATGCCTCTTTTTGTT
 2 An.sp.F_MK047669 100.0%  98.9%   TAGGGGCAGTAAATTTTATTACTACTGTCATTAATATACGATCTCCTGGAATTACATTAGATCGTATGCCTCTTTTTGTT
 3 An.sp.C_MK047666 100.0%  99.1%   TAGGGGCAGTAAATTTTATTACTACTGTCATTAATATACGATCTCCTGGAATTACATTAGATCGTATGCCTCTTTTTGTT
 4 An.sp.17_H01     100.0%  98.6%   TAGGGGCAGTAAATTTTATTACTACTGTCATTAATATACGATCTCCTGGAATTACATTAGATCGTATGCCTCTTTTTGTT
 5 An.sp.17_H02     100.0%  99.2%   TAGGGGCAGTAAATTTTATTACTACTGTCATTAATATACGATCTCCTGGAATTACATTAGATCGTATGCCTCTTTTTGTT
 6 An.sp.17_H03     100.0%  99.4%   TAGGGGCAGTAAATTTTATTACTACTGTCATTAATATACGATCTCCTGGAATTACATTAGATCGTATGCCTCTTTTTGTT
 7 An.sp.17_H04     100.0%  98.6%   TAGGGGCAGTAAATTTTATTACTACTGTCATTAATATACGATCTCCTGGAATTACATTAGATCGTATGCCTCTTTTTGTT
 8 An.sp.17_H05     100.0%  99.8%   TAGGGGCAGTAAATTTTATTACTACTGTCATTAATATACGATCTCCTGGAATTACATTAGATCGTATACCTCTTTTTGTT
 9 An.sp.17_H06     100.0%  99.1%   TAGGGGCAGTAAATTTTATTACTACTGTCATTAATATACGATCTCCTGGAATTACATTAGATCGTATGCCTCTTTTTGTT
10 An.sp.17_H07     100.0%  99.4%   TAGGGGCAGTAAATTTTATTACTACTGTCATTAATATACGATCTCCTGGAATTACATTAGATCGTATGCCTCTTTTTGTT
11 An.sp.17_H08     100.0%  99.7%   TAGGGGCAGTAAATTTTATTACTACTGTCATTAATATACGATCTCCTGGAATTACATTAGATCGTATGCCTCTTTTTGTT
12 An.sp.17_H09     100.0%  99.5%   TAGGGGCAGTAAATTTTATTACTACTGTCATTAATATACGATCTCCTGGAATTACATTAGATCGTATGCCTCTTTTTGTT
13 An.sp.17_H10     100.0%  99.5%   TAGGGGCAGTAAATTTTATTACTACTGTCATTAATATACGATCTCCTGGAATTACATTAGATCGTATGCCTCTTTTTGTT
14 An.sp.17_H11     100.0%  98.9%   TAGGGGCAGTAAATTTTATTACTACTGTCATTAATATACGATCTCCTGGAATTACATTAGATCGTATACCTCTTTTTGTT
15 An.sp.17_H12     100.0%  99.5%   TAGGGGCAGTAAATTTTATTACTACTGTCATTAATATACGATCTCCTGGAATTACATTAGATCGTATGCCTCTTTTTGTT
16 An.sp.17_H13     100.0%  98.9%   TAGGGGCAGTAAATTTTATTACTACTGTCATTAATATACGATCTCCTGGAATTACATTAGATCGTATGCCTCTTTTTGTT

                     cov    pid 481          .         5         .         .         .         .         :         . 560
 1 An.sp.D_MK047667 100.0% 100.0%   TGATCAGTAGTAATTACTGCTATTCTTTTATTACTTTCTTTACCTGTTTTAGCTGGAGCTATTACTATATTATTAACAGA
 2 An.sp.F_MK047669 100.0%  98.9%   TGATCAGTAGTAATTACTGCTATTCTTTTATTACTTTCTTTACCTGTTTTAGCTGGAGCTATTACTATATTATTAACAGA
 3 An.sp.C_MK047666 100.0%  99.1%   TGATCAGTGGTAATTACTGCTATTCTTTTATTACTTTCTTTACCTGTTTTAGCTGGAGCTATTACTATATTATTAACAGA
 4 An.sp.17_H01     100.0%  98.6%   TGATCAGTAGTAATTACTGCTATTCTTTTATTACTTTCTTTACCTGTTTTAGCTGGAGCTATTACTATATTATTAACAGA
 5 An.sp.17_H02     100.0%  99.2%   TGATCAGTAGTAATTACTGCTATTCTTTTATTACTTTCTTTACCTGTTTTAGCTGGAGCTATTACTATATTATTAACAGA
 6 An.sp.17_H03     100.0%  99.4%   TGATCAGTAGTAATTACTGCTATTCTTTTATTACTTTCTTTACCTGTTTTAGCTGGAGCTATTACTATATTATTAACAGA
 7 An.sp.17_H04     100.0%  98.6%   TGATCAGTAGTAATTACTGCTATTCTTTTATTACTTTCTTTACCTGTTTTAGCTGGAGCTATTACTATATTATTAACAGA
 8 An.sp.17_H05     100.0%  99.8%   TGATCAGTAGTAATTACTGCTATTCTTTTATTACTTTCTTTACCTGTTTTAGCTGGAGCTATTACTATATTATTAACAGA
 9 An.sp.17_H06     100.0%  99.1%   TGATCAGTAGTAATTACTGCTATTCTTTTATTACTTTCTTTACCTGTTTTAGCTGGAGCTATTACTATATTATTAACAGA
10 An.sp.17_H07     100.0%  99.4%   TGATCAGTAGTAATTACTGCTATTCTTTTATTACTTTCTTTACCTGTTTTAGCTGGAGCTATTACTATATTATTAACAGA
11 An.sp.17_H08     100.0%  99.7%   TGATCAGTAGTAATTACTGCTATTCTTTTATTACTTTCTTTACCTGTTTTAGCTGGAGCTATTACTATATTATTAACAGA
12 An.sp.17_H09     100.0%  99.5%   TGATCAGTAGTAATTACTGCTATTCTTTTATTACTTTCTTTACCTGTTTTAGCTGGAGCTATTACTATATTATTAACAGA
13 An.sp.17_H10     100.0%  99.5%   TGATCAGTAGTAATTACTGCTATTCTTTTATTACTTTCTTTACCTGTTTTAGCTGGAGCTATTACTATATTATTAACAGA
14 An.sp.17_H11     100.0%  98.9%   TGATCAGTAGTAATTACTGCTATTCTTTTATTACTTTCTTTACCTGTTTTAGCTGGAGCTATTACTATATTATTAACAGA
15 An.sp.17_H12     100.0%  99.5%   TGATCAGTAGTAATTACTGCTATTCTTTTATTACTTTCTTTACCTGTTTTAGCTGGAGCTATTACTATATTATTAACAGA
16 An.sp.17_H13     100.0%  98.9%   TGATCAGTAGTAATTACTGCTATTCTTTTATTACTTTCTTTACCTGTTTTAGCTGGAGCTATTACTATATTATTAACAGA

                     cov    pid 561          .         .         .         6         .         .         .     ] 636
 1 An.sp.D_MK047667 100.0% 100.0%   TCGAAATTTAAATACATCTTTCTTTGATCCTGCTGGAGGAGGAGACCCTATTTTATACCAACACTTATTTTGATTT
 2 An.sp.F_MK047669 100.0%  98.9%   TCGAAATTTAAATACATCTTTTTTTGATCCTGCTGGAGGAGGAGACCCTATTTTATACCAACACTTATTTTGATTT
 3 An.sp.C_MK047666 100.0%  99.1%   TCGAAATTTAAATACATCTTTCTTTGATCCTGCTGGAGGAGGAGATCCTATTTTATACCAACACTTATTTTGATTT
 4 An.sp.17_H01     100.0%  98.6%   TCGAAATTTAAATACATCTTTCTTTGATCCTGCCGGGGGAGGGGATCCTATTTTATACCAACACTTATTTTGATTT
 5 An.sp.17_H02     100.0%  99.2%   TCGAAATTTAAATACATCTTTCTTTGATCCTGCTGGGGGAGGGGATCCTATTTTATACCAACACTTATTTTGATTT
 6 An.sp.17_H03     100.0%  99.4%   TCGAAATTTAAATACATCTTTCTTTGATCCTGCTGGAGGAGGGGATCCTATTTTATACCAACACTTATTTTGATTT
 7 An.sp.17_H04     100.0%  98.6%   TCGAAATTTAAATACATCTTTCTTTGATCCTGCTGGGGGAGGGGATCCTATTTTATACCAACACTTATTTTGATTT
 8 An.sp.17_H05     100.0%  99.8%   TCGAAATTTAAATACATCTTTCTTTGATCCTGCTGGAGGAGGAGACCCTATTTTATACCAACACTTATTTTGATTT
 9 An.sp.17_H06     100.0%  99.1%   TCGAAATTTAAATACATCTTTCTTTGATCCTGCTGGGGGAGGGGATCCTATTTTATACCAACACTTATTTTGATTT
10 An.sp.17_H07     100.0%  99.4%   TCGAAATTTAAATACATCTTTCTTTGATCCTGCTGGAGGAGGGGATCCTATTTTATACCAACACTTATTTTGATTT
11 An.sp.17_H08     100.0%  99.7%   TCGAAATTTAAATACATCTTTCTTTGATCCTGCTGGAGGAGGGGACCCTATTTTATACCAACACTTATTTTGATTT
12 An.sp.17_H09     100.0%  99.5%   TCGAAATTTAAATACATCTTTCTTTGATCCTGCTGGAGGAGGGGATCCTATTTTATACCAACACTTATTTTGATTT
13 An.sp.17_H10     100.0%  99.5%   TCGAAATTTAAATACATCTTTCTTTGATCCTGCTGGAGGAGGGGATCCTATTTTATACCAACACTTATTTTGATTT
14 An.sp.17_H11     100.0%  98.9%   TCGAAATTTAAATACATCTTTCTTTGATCCTGCTGGAGGAGGGGATCCTATTTTATACCAACACTTATTTTGATTT
15 An.sp.17_H12     100.0%  99.5%   TCGAAATTTAAATACATCTTTCTTTGATCCTGCTGGAGGAGGGGATCCTATTTTATACCAACACTTATTTTGATTT
16 An.sp.17_H13     100.0%  98.9%   TCGAAATTTAAATACATCTTTCTTTGATCCTGCTGGAGGAGGGGATCCTATTTTATACCAACACTTATTTTGATTT
```
